# Enhanced replication and immunogenicity of a genome-modified non-replicating vaccinia virus Tiantan strain for robust low-dose protection in mice

**DOI:** 10.1128/jvi.00943-26

**Published:** 2026-07-31

**Authors:** Jiao Ren, Shiyuan Liu, Hang Yuan, Yiyi Wu, Zhihao Zhao, Yawei Wang, Baoying Huang, Wenling Wang, Houwen Tian, Wenjie Tan

**Affiliations:** 1 National Key Laboratory of Intelligent Tracking and Forecasting for Infectious Diseases, Key Laboratory of Biosafety, National Health Commissions, National Institute for Viral Disease Control and Prevention, China CDC 829687 , , Beijing, People's Republic of China; Northwestern University Feinberg School of Medicine, Chicago, Illinois, USA

**Keywords:** vaccinia virus, NTV-ΔF1L-C7L, monkeypox virus (MPXV), recombinant virus, F1L deletion, vaccine, immunogenicity

## Abstract

A non-replicating Tiantan strain-based vaccinia virus (NTV) holds significant application prospects for vaccination and gene therapy and has recently entered clinical trials as a novel and safer vaccine candidate against monkeypox. However, optimization is still required, particularly regarding its production capacity and immunogenicity. In this study, a recombinant virus was constructed by modifying the F1L and C7L genes in a non-replicating viral backbone using CRISPR/Cas9-mediated gene editing and homologous recombination. The resulting construct, designated NTV-ΔF1L-C7L, exhibited significantly enhanced replication in vaccine production cell lines, with viral yields increasing by more than 680-fold in MRC-5 cells compared to those of the parental NTV. Its pathogenicity in mice was significantly reduced, showing more than a 10-fold decrease compared to the pathogenicity of the vaccinia virus Tiantan strain (VTT). Following two intramuscular doses, NTV-ΔF1L-C7L elicited high titers of orthopoxvirus-specific IgG and neutralizing antibodies against vaccinia and monkeypox viruses, along with a robust cellular immune response exhibiting a Th1 bias, which was significantly stronger than that induced by either parental NTV or VTT vaccination. Complete protection (100%) against a lethal challenge with the vaccinia virus Western Reserve strain was achieved in mice immunized with either a low dose (10^3^ PFU) or a single dose (10⁵ PFU) of NTV-ΔF1L-C7L, comparable to that conferred by VTT. These findings demonstrate that NTV-ΔF1L-C7L combines high safety with enhanced replication and immunogenicity, supporting its value as a novel vaccine vector and its potential application in controlling the current global monkeypox outbreak.

**IMPORTANCE:**

A highly attenuated NTV exhibits an improved safety profile; however, its production capacity and immunogenicity require optimization for clinical application. In this study, a novel recombinant virus, NTV-ΔF1L-C7L, was developed by targeting the deletion of F1L and the insertion of C7L into the NTV backbone. This attenuated live vaccine, NTV-ΔF1L-C7L, demonstrates several significant advantages. Its robust *in vitro* replication supports scalability for large-scale vaccine production. It has demonstrated strong immunoprotective efficacy in mice while maintaining a high safety margin, exhibiting reduced pathogenicity *in vivo* , and inducing robust humoral and cellular immune responses against vaccinia and monkeypox virus. These immune responses confer complete protection against lethal VACV challenge at low-dose or single-dose immunization. These findings establish a solid experimental foundation for the further development of NTV-ΔF1L-C7L as a next-generation VACV-based vector or a candidate vaccine against mpox.

## INTRODUCTION

Vaccinia virus (VACV), a member of the *Orthopoxvirus* genus, is a large double-stranded DNA virus with a genome of approximately 195 kb (1). VACV played a crucial role in the global eradication of smallpox (2), contributing to the World Health Organization’s (WHO) declaration of its eradication in 1980 (3). In the post-eradication era, VACV has served as a model for elucidating virus–host interactions and has been widely used to develop vaccine vectors and oncolytic virotherapy owing to its broad host tropism, high transgene expression efficiency, and strong immunogenicity (4–6). Despite their effectiveness, VACV-based vaccines have been associated with adverse effects, including local inflammation, pain, and swelling, as well as rare, severe systemic complications, such as progressive vaccinia and myocarditis (7, 8). To mitigate these risks, several attenuated VACV strains have been developed through targeted genetic modification, with modified vaccinia virus Ankara (MVA) being among the most extensively studied. These attenuated strains exhibit significantly reduced virulence, expanding their applications in prophylactic and therapeutic contexts (9, 10). These have been used to develop vaccines against monkeypox (mpox) (11). Following the global mpox outbreak declared in 2022, 150,092 confirmed cases and 343 deaths had been reported across 137 countries and regions as of 31 May 2025 (12). Three WHO-approved vaccines, ACAM2000, JYNNEOS, and LC16m8, are currently available, all derived from attenuated VACV strains originally developed for smallpox prevention. Owing to the over 90% genome sequence homology between VACV and monkeypox virus (MPXV), these vaccines provide cross-protection, with an efficacy exceeding 85% (13). This protection is primarily mediated by the induction of neutralizing antibodies and robust T-cell responses. However, each vaccine has limitations: ACAM2000 is associated with significant adverse events (14), JYNNEOS is constrained by limited production capacity (15), and LC16m8 requires scarification for administration, which limits its scalability for mass immunization (16).

In China, the vaccinia virus Tiantan strain (VTT) was used extensively during the smallpox eradication campaign and remains a backbone in viral vector and gene therapy research (7, 17, 18). However, its high virulence restricts its direct use. To overcome this limitation, we previously engineered a highly attenuated, non-replicating Tiantan strain-based vaccinia virus (NTV) by deleting 26 genes involved in host range and virulence (19). NTV has an improved safety profile and has received clinical trial approval from the National Medical Products Administration of China as a candidate MPXV vaccine (20). Nevertheless, NTV exhibits reduced production capacity and weaker immunogenicity compared to VTT (21), necessitating more complex production and higher immunization doses. Unpublished data from our laboratory also indicate that NTV induces weaker immune responses than MVA at equivalent doses. Thus, further genetic optimization of NTV is required.

A widely used strategy involves the deletion or modification of viral immune evasion genes (22–24), as exemplified by the development of MVA and NYVAC, a highly attenuated VACV strain derived from the Copenhagen vaccine strain through the precise deletion of 18 open reading frames from the viral genome (25). The C7L gene, a host range factor identified through comparative genomic analysis between MVA and NYVAC (26), regulates late gene expression and contributes to the superior immunogenicity of MVA (27). Its reintroduction into NYVAC restores late protein synthesis (28). Given the biological parallels between NTV and NYVAC, C7L insertion into NTV may similarly improve immunogenicity. Although C7L is not directly associated with virulence (28), its impact on the viral phenotype necessitates careful evaluation. Anti-apoptotic genes have been implicated in the regulation of VACV virulence and host immunity (29). Genomic analysis of NTV identified E3L and F1L as key anti-apoptotic genes. E3L is essential for intermediate and late gene expression, and its deletion significantly impairs immunogenicity (30, 31). F1L encodes a conserved tail-anchored protein that localizes to the mitochondrial outer membrane and inhibits apoptosis by stabilizing mitochondrial membrane potential (32). Deletion of F1L enhances apoptosis, attenuates virulence (33), and upregulates the expression of type I interferons (IFN-α and IFN-β), pro-inflammatory cytokines (e.g., IL-1β, TNF, IL-6, IL-12p40), and chemokines (e.g., MIP-1α) (34). F1L-deficient VACV also enhances CD8^+^ T -cell responses, characterized by increased magnitude and shortened contraction phases (34), supporting a dual role for F1L deletion in improving both safety and immunogenicity. These limitations underscore the need for further optimization of non-replicating vaccinia vectors to enhance immunogenicity and productivity.

This study aimed to construct a recombinant virus, NTV-ΔF1L-C7L, by deleting F1L and inserting *C7L* into the NTV backbone. Its *in vitro* replication kinetics, *in vivo* safety, and immunogenicity were systematically evaluated, with particular emphasis on protective efficacy following either low-dose (10³ PFU) or single-dose (10⁵ PFU) immunization. Our findings demonstrate that NTV-ΔF1L-C7L exhibits enhanced replication *in vitro* , reduced pathogenicity *in vivo* , and robust humoral and cellular immune responses, conferring complete protection against lethal VACV challenge after low- or single-dose immunization.

## RESULTS

### NTV-ΔF1L-C7L restores MPXV-cross-reactive antigen expression and triggers apoptosis

**Fig 1:**
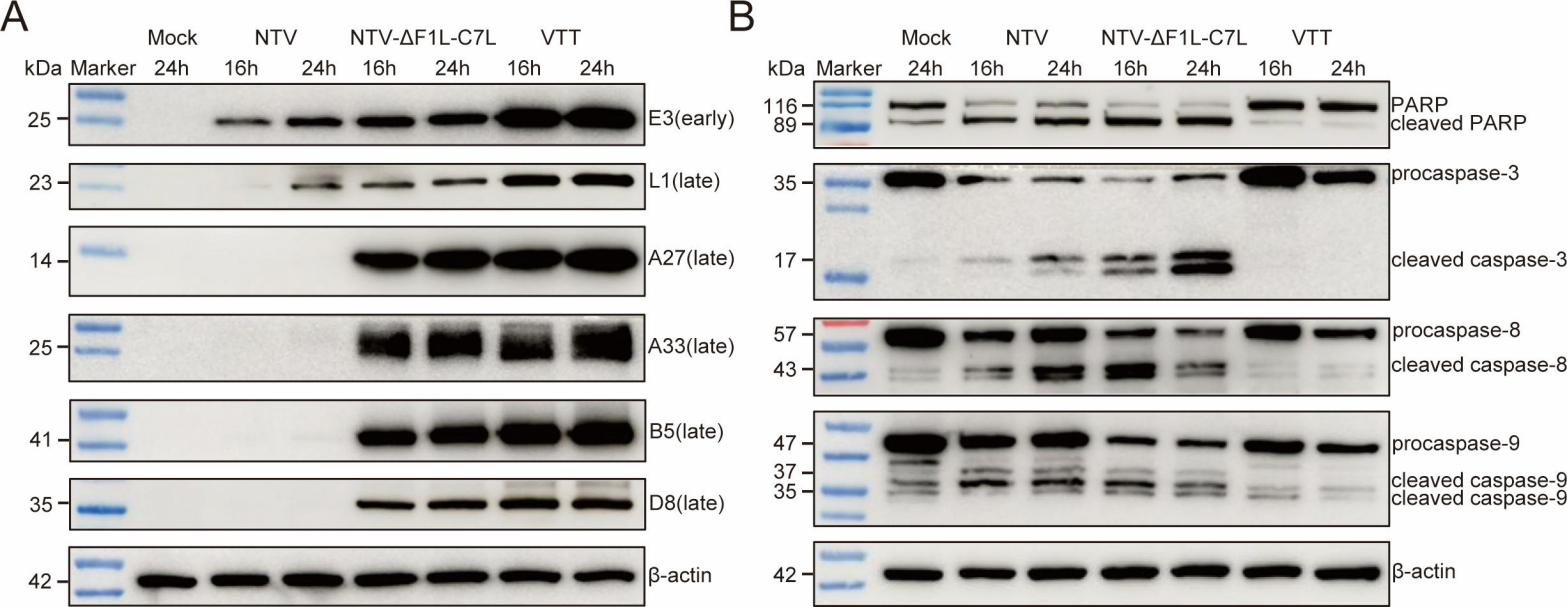
Expression of VACV proteins antigenically cross-reactive with MPXV antigens and detection of virus-induced apoptotic molecular changes. HeLa cell monolayers were mock-infected or infected at a multiplicity of infection (MOI) of 5 with NTV, NTV-ΔF1L-C7L, or VTT . Cell lysates were harvested at 16 h and 24 h post-infection and analyzed using western blot with (**A**) MPXV -specific antibodies to detect cross-reactive VACV proteins: early protein E3 (OPG065; cross-reactive with MPXV E3) and late proteins L1 (OPG099; MPXV M1), A27 (OPG154; MPXV A29), A33 (OPG161; MPXV A35), B5 (OPG190; MPXV B6), and D8 (OPG105; MPXV E8) or (**B**) antibodies against apoptosis-related factors. Apoptosis induction was assessed by detecting cleavage of key apoptotic markers: Poly (ADP-ribose) polymerase (PARP), a DNA repair enzyme whose cleavage by caspases serves as a hallmark of irreversible apoptosis; caspase-3, the primary executioner caspase responsible for proteolytic cleavage of cellular substrates during apoptosis; caspase-8, the initiator caspase of the extrinsic (death receptor-mediated) apoptotic pathway; and caspase-9, the initiator caspase of the intrinsic (mitochondrial) apoptotic pathway. Cell apoptosis leads to the cleavage of full-length PARP (116 kDa) into cleaved PARP (89 kDa), procaspase-3 (35 kDa) into cleaved caspase-3 (17 kDa), procaspase-8 (57 kDa) into cleaved caspase-8 (43 kDa), and procaspase-9 (47 kDa) into cleaved caspase-9 (37 and 35 kDa).

HeLa cells were subjected to western blot analysis at 16 and 24 h post-infection (hpi) to evaluate the effects of VTT, NTV, or NTV-ΔF1L-C7L infection on the expression of proteins antigenically cross-reactive with MPXV and host apoptotic responses. As shown in Fig. 1A, the VACV early protein E3 (OPG065; antigenically cross-reactive with MPXV E3) was stably expressed in all three groups at both 16 and 24 h. VTT robustly expressed all five VACV late proteins, L1 (OPG099; MPXV M1), A27 (OPG154; MPXV A29), A33 (OPG161; MPXV A35), B5 (OPG190; MPXV B6), and D8 (OPG105; MPXV E8), which were antigenically cross-reactive with their MPXV counterparts at both 16 and 24 h. In contrast, the expression of these cross-reactive late proteins in the NTV group was markedly impaired; only L1 was detectable at 24 h, whereas the other four proteins (A27, A33, B5, and D8) were barely detectable at either time point. The NTV-ΔF1L-C7L group expressed all five cross-reactive late proteins as early as 16 h, with further enhancement at 24 h. In this group, L1 and B5 were consistently expressed at levels slightly lower than those of the VTT group, whereas A27, A33, and D8 reached expression levels comparable to those in the VTT group.

Apoptosis induction was assessed by detecting the cleavage of key apoptotic markers (Fig. 1B): PARP, a DNA repair enzyme whose cleavage by caspases serves as a hallmark of irreversible apoptosis; caspase-3, the primary executioner caspase responsible for the proteolytic cleavage of cellular substrates during apoptosis; caspase-8, the initiator caspase of the extrinsic (death receptor-mediated) apoptotic pathway; and caspase-9, the initiator caspase of the intrinsic (mitochondrial) apoptotic pathway. As shown in Fig. 1B, cleaved PARP was detectable in all three infection groups at 16 h, with the strongest signal in the NTV-ΔF1L-C7L group, an intermediate level in the NTV group, and the weakest in the VTT group; this difference became more pronounced at 24 h. Cleaved caspase-3 was observed at 16 h in both NTV and NTV-ΔF1L-C7L groups (the latter exhibiting a stronger signal), whereas only a faint band was detected in the VTT group. By 24 h, cleaved caspase-3 signals intensified in both NTV and NTV-ΔF1L-C7L groups, whereas the VTT group had nearly undetectable signals. Cleaved caspase-8 and cleaved caspase-9 were also detectable at 16 h in the NTV and NTV-ΔF1L-C7L groups. At 24 h, the signals for both caspases decreased in the NTV-ΔF1L-C7L group but increased in the NTV group, whereas the VTT group displayed consistently weak signals at both time points.

### NTV-ΔF1L-C7L demonstrates robust cell-to-cell spread across multiple cell lines

**Fig 2:**
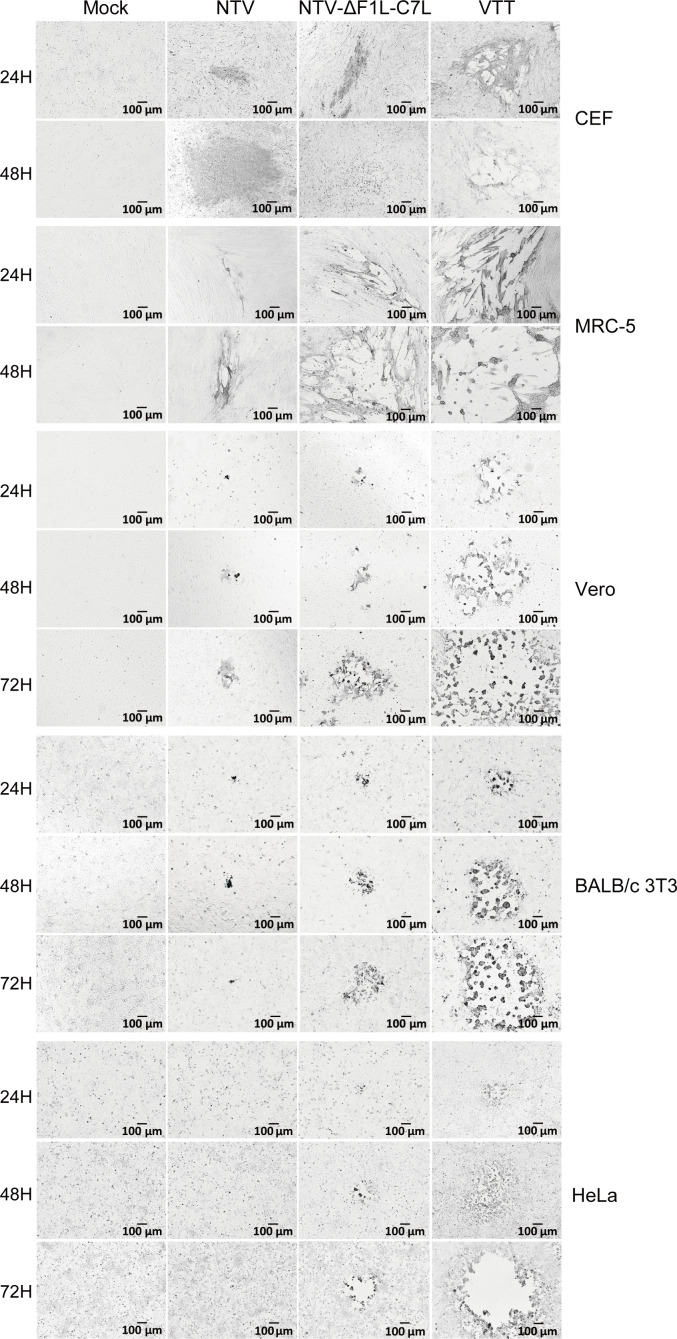
Cell-to-cell transmission of NTV-ΔF1L-C7L. Cells were infected with the virus at an MOI of 0.005, fixed at 24, 48, or 72 hpi, and stained with anti-VACV antibody, followed by plaque visualization using TrueBlue peroxidase substrate. NTV and VTT were used as controls. All images were captured at a magnification of 40×.

CEF, MRC-5, Vero, BALB/c 3T3, and HeLa cells were infected at an MOI of 0.005 and then immunohistochemically stained with anti-VACV serum at 24, 48, and 72 hpi to visualize plaque formation and evaluate the cell-to-cell spread capacities of NTV, NTV-ΔF1L-C7L, and VTT (Fig. 2). Based on plaque size and lesion morphology, a semi-quantitative assessment of viral spread in each cell line was conducted (Table 1).

**TABLE 1:** Replication and spread of NTV-ΔF1L-C7L in five cell types (based on multi-step growth curves)

Cell line	Species	Virus spread^*a*^						Virus replication^*b*^					
		48 h			72 h			48 h			72 h		
		NTV	NTV- ΔF1L-C7L	VTT	NTV	NTV- ΔF1L-C7L	VTT	NTV	NTV- ΔF1L-C7L	VTT	NTV	NTV- ΔF1L-C7L	VTT
CEF	Chicken embryo	+++	++++	+++	++++	++++	+++	1221.37 (P)	4699.35 (P)	863.28 (P)	2354.54 (P)	37969.24 (P)	1127.11 (P)
MRC-5	Human	-	++++	++++	-	++++	++++	2.50 (SP)	896.96 (P)	3874.64 (P)	2.91 (SP)	1973.75 (P)	7189.04 (P)
Vero	African green monkey	-	+	++	-	++	++++	0.61 (NP)	5.55 (SP)	17.74 (SP)	0.99 (NP)	45.99 (P)	447.98 (P)
BALB/c 3T3	Mouse	-	+	++	-	+	+++	1.28 (SP)	65.67 (P)	2188.52 (P)	0.63 (NP)	437.27 (P)	7316.02 (P)
HeLa	Human	-	+	++	-	+	+++	0.12 (NP)	153.63 (P)	1374.23 (P)	0.1 (NP)	400.52 (P)	1993.87 (P)

^*a*^ Virus spread (virus plaque area as a percentage of area under the field of view) as visualized by immunostaining after 48 or 72 h. -, <5%; +, 5% to 25% ; ++, 25% to 50%; +++, 50% to 80%; ++++, >80%.

^*b*^ Virus replication (fold increase in virus titer) determined by dividing the virus yield at 48 or 72 h by the practical input titer. Cell lines were classified into permissive (P, >25-fold increase), semi-permissive (SP, 1- to 25-fold increase), and non-permissive (NP, <1-fold increase) cells.

In CEF cells, both NTV and NTV-ΔF1L-C7L formed clearly visible stained foci at 24 and 48 hpi, with progressive expansion over time and no significant cell detachment. In contrast, VTT induced partial cell rounding and detachment as early as 24 hpi, progressing to widespread monolayer disruption by 48 hpi, characteristic of classical cytopathic effect. The plaque sizes of NTV-ΔF1L-C7L and NTV were larger than those of VTT, indicating superior intercellular spread in this cell line. In MRC-5 cells, NTV produced only small, sparse foci (typically fewer than five stained cells). NTV-ΔF1L-C7L formed substantially larger lesions by 48 hpi, with plaque sizes approaching those of VTT, demonstrating markedly enhanced spreading capacity. VTT initiated visible plaque formation by 24 hpi and expanded into large, dense lesions by 48 hpi. In Vero cells, NTV continued to exhibit only weakly stained foci. NTV-ΔF1L-C7L showed delayed plaque development, with detectable lesions primarily at 72 hpi and smaller than those of VTT. In contrast, VTT formed distinct plaques by 48 hpi, which continued to expand through 72 hpi, reflecting robust spreading ability. In BALB/c 3T3 and HeLa cells, the final plaque sizes among the three viruses were comparable; however, their spreading kinetics differed markedly. VTT displayed a clear trend of progressive expansion over time, whereas NTV-ΔF1L-C7L spread more slowly, and NTV barely formed visible plaques or showed any discernible dissemination.

Collectively, based on the semi-quantitative scoring of viral spread in each cell line (Table 1), we observed a clear cell type-dependent pattern: in CEF cells, both NTV-ΔF1L-C7L and NTV exhibited greater spreading capacity than VTT, whereas in MRC-5, Vero, BALB/c 3T3, and HeLa cells, the spreading efficiency followed the order VTT > NTV-ΔF1L-C7L > NTV.

### NTV-ΔF1L-C7L supports efficient multi-cycle replication across diverse cell lines

**Fig 3:**
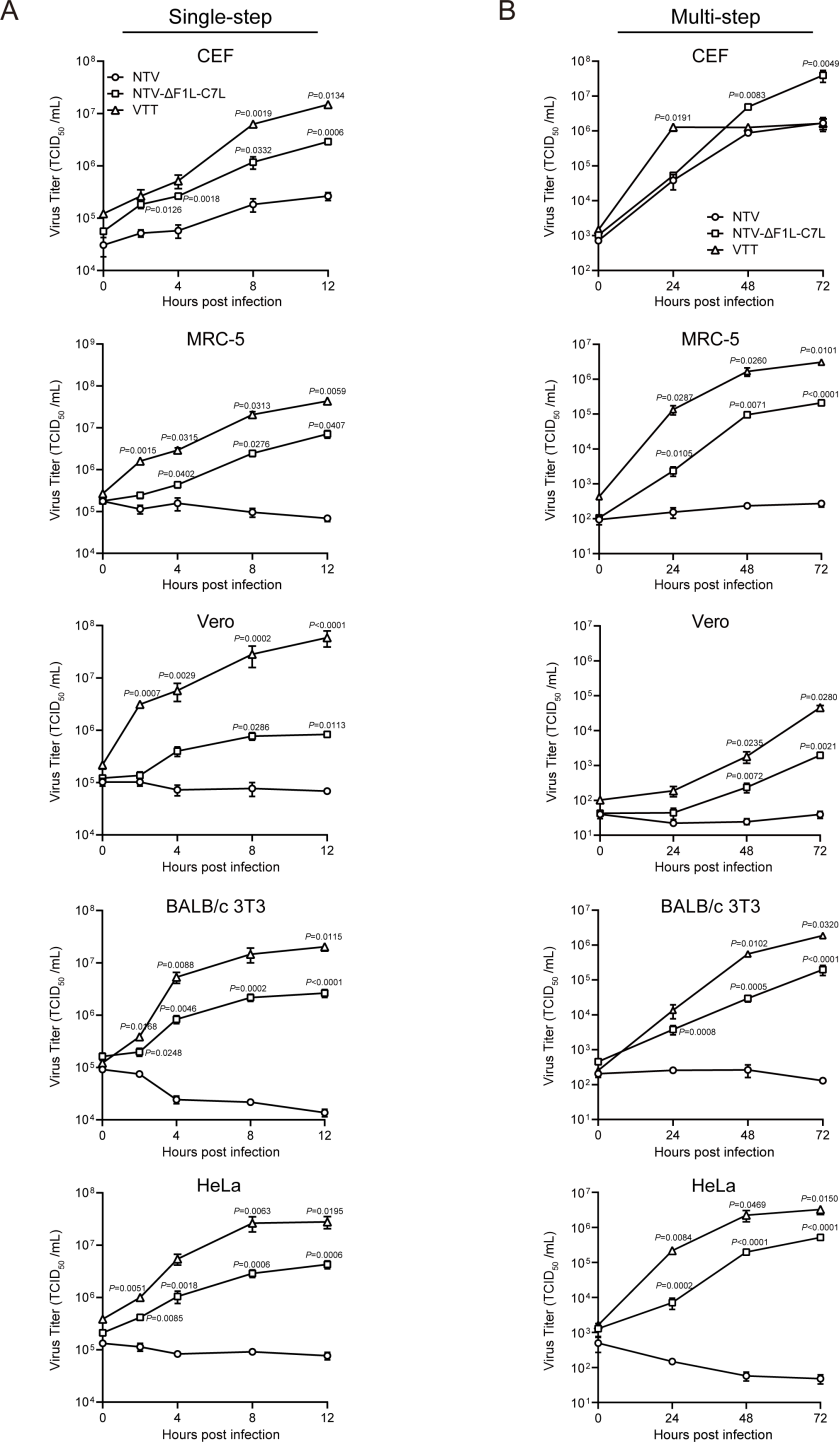
Replication of NTV-ΔF1L-C7L. (**A and B**) Single-step and multi-step growth curve. For the single-step growth curve, cells were infected with the virus at an MOI of 5 and harvested at 0, 2, 4, 8, and 12 hpi. For the multi-step growth curve, cells were infected with the virus at an MOI of 0.01, harvested at 0, 24, 48, and 72 hpi, and all samples were subjected to three cycles of freeze–thaw, followed by TCID_50_ titration of virus titer using BHK-21 cells. NTV and VTT were used as controls. The data were obtained from three independent replicate experiments, and viral growth kinetics curves were plotted using GraphPad Prism 10.1.2. Data are reported as mean ± standard error of the mean (SEM). Statistical significance was assessed using one-way analysis of variance (ANOVA) with multiple comparisons test. For clarity, *P*-values are indicated directly above the corresponding curves as follows: the *P*-value above the VTT curve represents comparison with NTV-ΔF1L-C7L; the *P*-value above the NTV-ΔF1L-C7L curve represents comparison with NTV. A *P*-value of less than 0.05 was considered statistically significant.

Single-step and multi-step growth assays were performed in the five cell lines to evaluate the replication kinetics of NTV-ΔF1L-C7L. In single-step infections (MOI = 5), NTV-ΔF1L-C7L initiated productive virus production as early as 4 hpi in all tested cell types (Fig. 3A). Viral titers plateaued between 8 and 12 hpi, with the highest yields observed in CEF and MRC-5 cells. In contrast, BALB/c 3T3, Vero, and HeLa cells supported only moderate early replication, with viral titers at 12 hpi increasing by less than one log₁₀ relative to the input inoculum. In multi-step growth curves (MOI = 0.01), NTV-ΔF1L-C7L underwent efficient multi-cycle replication over 72 h, with kinetics and final yields exhibiting strong cell type dependence (Fig. 3B). CEF cells supported the most robust amplification, achieving a >10⁴-fold increase in viral titer at 72 hpi. MRC-5 cells also permitted efficient replication, with a >10³-fold titer increase. BALB/c 3T3 and HeLa cells displayed intermediate replication capacity, yielding modest increases of approximately 10²-fold over the course of infection. In Vero cells, replication was slow and limited, resulting in only a marginal titer increase by 72 hpi. The parental VTT strain also exhibited reduced replication efficiency in this cell line.

These results are consistent with those of replication efficiency assessment in Table 1, which classifies cellular permissiveness for NTV-ΔF1L-C7L based on fold changes in viral titer at 48 and 72 hpi according to established poxvirus criteria (35). CEF, MRC-5, BALB/c 3T3, and HeLa cells were designated as fully permissive (P) at both time points, indicating sustained and high-yield replication. Vero cells were semi-permissive (SP) at 48 hpi but transitioned to permissive (P) at 72 hpi, reflecting delayed but productive infection. These data demonstrate that NTV-ΔF1L-C7L has regained efficient multi-cycle replication capacity across a range of mammalian and avian cell lines.

### NTV-ΔF1L-C7L exhibits favorable safety characteristics

**Fig 4:**
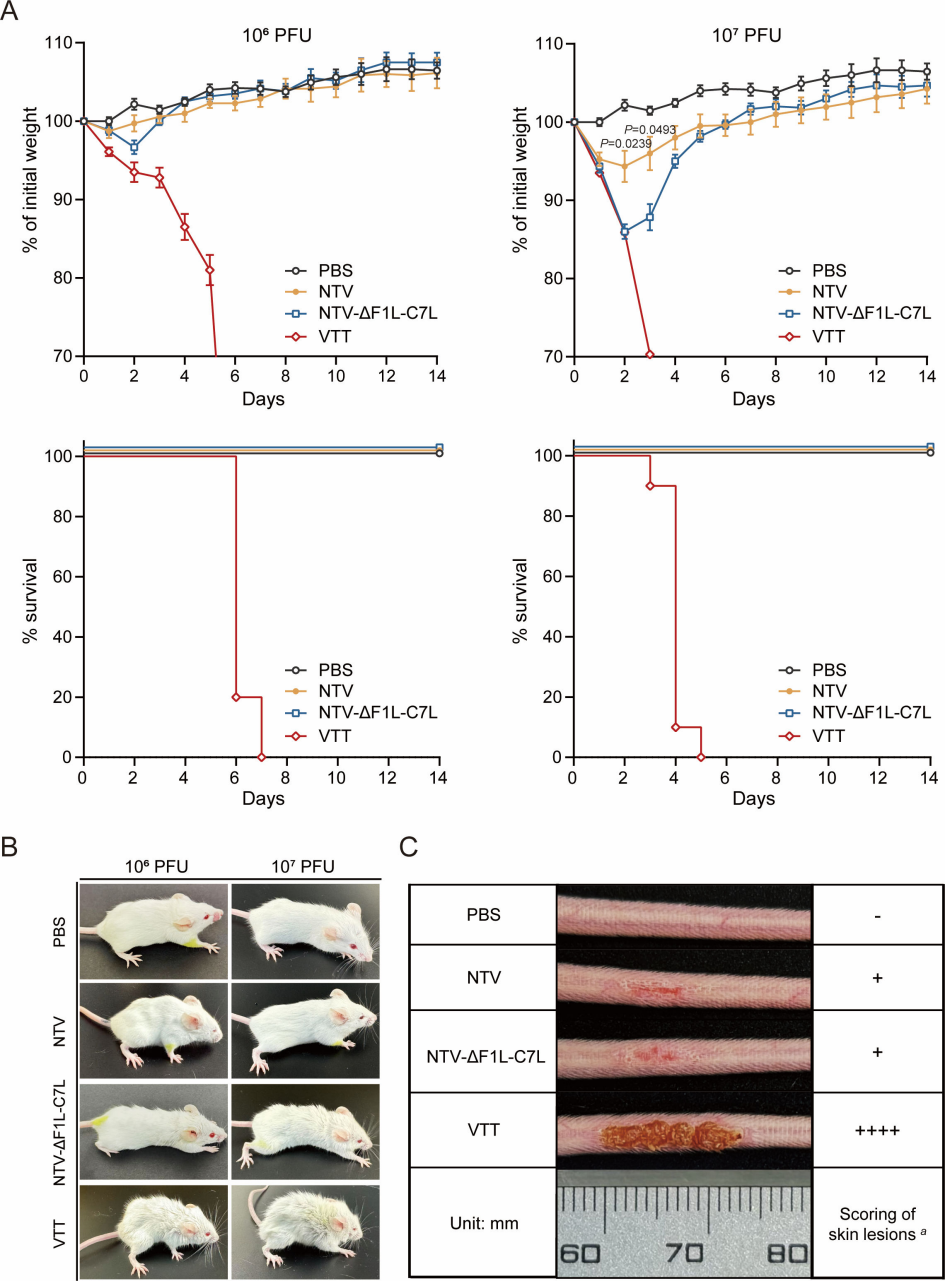
Virulence of NTV-ΔF1L-C7L. (**A**) Virulence in an upper respiratory tract infection model. Six -week-old female BALB/c mice (*n* = 10) were intranasally infected with 10^6^ and 10^7^ PFU of NTV, NTV-ΔF1L-C7L, and VTT viruses. The body weight control group was mock-infected with PBS. The animals were monitored daily for body weight loss and survival for 14 days. Error bars represent the SEM. On the body weight change curve for the 10⁷ PFU intranasal challenge, statistical comparisons between NTV and NTV-ΔF1L-C7L on days 2 and 3 are indicated directly above the curves, with *P*-values shown to denote significant differences. (**B**) Mouse signs were photographed at 2 dpi with the three viruses, with the PBS group as the negative control. (**C**) Lesion formation after tail scarification. Groups of 5-week-old BALB/c mice (*n* = 3) were tail-scarified with 10^7^ PFU of NTV and NTV-ΔF1L-C7L or 2 × 10^5^ PFU of VTT using a bifurcated needle. The control group was mock-inoculated with PBS in the same manner. The development of tail lesions in mice was monitored daily. Images were captured at 12 dpi. Lesion scores were categorized as follows: -, no visible lesions; +, mild erythema and formation of bloody wounds; ++, mild erythema and visible scabbing; +++, minor scabbing and mild skin ulceration; and ++++, visible significant scabbing and more severe skin ulceration.

The *in vivo* virulence of NTV-ΔF1L-C7L was evaluated in 6-week-old female BALB/c mice (*n* = 10 per group) following intranasal inoculation with 10⁶ or 10⁷ PFU/30 μL of NTV-ΔF1L-C7L, NTV, or VTT. Clinical signs, body weight changes, and survival were monitored daily for 14 days. At the 10⁶ PFU dose, all VTT-infected mice exhibited progressive weight loss (Fig. 4A), with clinical signs of acute infection—including reduced activity, piloerection, and lethargy—evident by 2 days post-infection (dpi) (Fig. 4B). By 6–7 dpi, these mice had lost >25% of their initial body weight and were euthanized in accordance with humane endpoints. In contrast, mice infected with either NTV or NTV-ΔF1L-C7L showed minimal body weight fluctuations (<4%) and no overt clinical abnormalities throughout the observation period, comparable to phosphate-buffered saline (PBS)-treated controls, indicating a favorable safety profile at this dose. At the higher dose (10⁷ PFU), differential virulence was observed across all groups. VTT induced the most severe disease, resulting in >30% wt loss and 100% mortality by 5 dpi. Mice displayed marked signs of illness—including hunched posture, severe piloerection, and impaired motor coordination—as early as 2 dpi. In contrast, NTV-ΔF1L-C7L caused only transient weight loss (~14% at 2 dpi) and mild piloerection in a subset of animals, with rapid recovery beginning at 3 dpi. Mice infected with NTV exhibited negligible weight change (<5%) and remained clinically indistinguishable from PBS controls. These results indicate that although NTV-ΔF1L-C7L displays slightly enhanced virulence relative to NTV at high doses, it remains markedly less pathogenic than VTT—even when administered at a 10-fold lower inoculum (10⁶ PFU).

Cutaneous virulence was further assessed using tail scarification. Mice inoculated with 2 × 10⁵ PFU of VTT developed pronounced local inflammation and extensive scab formation (mean lesion length: 1.3 ± 0.2 cm) by 12 dpi (Fig. 4C). In stark contrast, mice receiving 10⁷ PFU of either NTV or NTV-ΔF1L-C7L exhibited only mild erythema and superficial epidermal disruption, with no scab formation observed. These findings confirm that NTV-ΔF1L-C7L possesses substantially attenuated cutaneous virulence compared to that of VTT.

### NTV-ΔF1L-C7L induces orthopoxvirus-specific IgG responses

**Fig 5:**
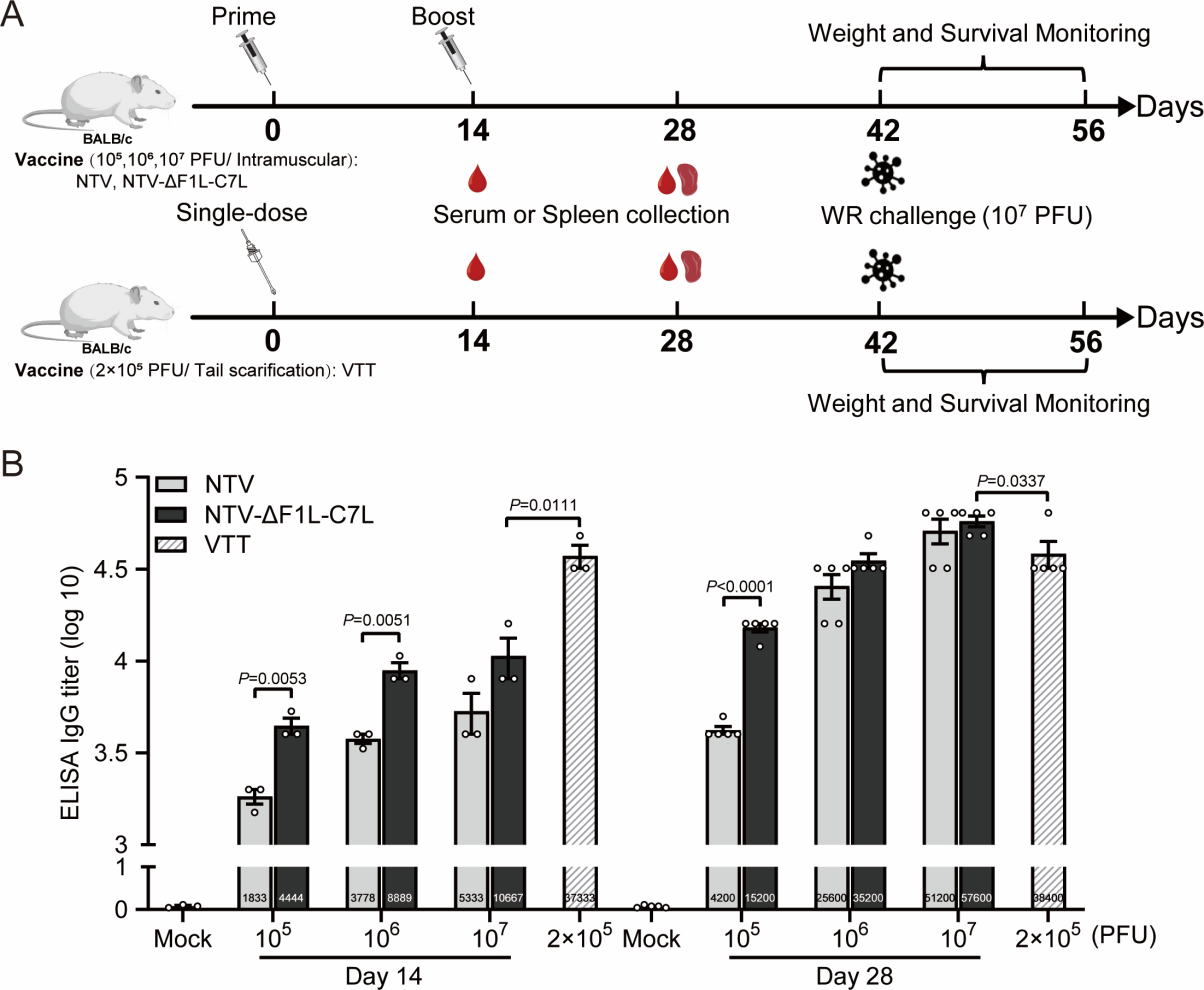
Immunization schedule and anti-VACV virion IgG responses. (**A**) Immunization schedule. Female 6-week-old BALB/c mice were randomly divided into groups (*n* = 21). Mice were immunized intramuscularly with 10⁵–10⁷ PFU of NTV or NTV-ΔF1L-C7L twice at a 2-week interval, or once with 2 × 10⁵ PFU of VTT via tail scarification. Serum and splenocyte samples were collected at the indicated time points. Two weeks after primary immunization, three mice per group were used for immunological analysis; 2 weeks after the booster, five mice per group were analyzed. The remaining mice were challenged with 10⁷ PFU (25× LD₅₀) of VACV Western Reserve (WR). (**B**) Anti-VACV virion IgG titers in mouse serum at 14 and 28 days after primary immunization, measured by ELISA. Data are shown as mean ± SEM. Statistical analysis was performed using one-way ANOVA with multiple comparisons. *P* -values for NTV-ΔF1L-C7L vs NTV and NTV-ΔF1L-C7L vs VTT are indicated directly above the corresponding bars.

BALB/c mice were intramuscularly immunized with three doses of NTV or NTV-ΔF1L-C7L (10⁵, 10⁶, or 10⁷ PFU), with a homologous booster on day 14, to evaluate the immunogenicity of NTV-ΔF1L-C7L (Fig. 5A). As a positive control, VTT was administered by tail scarification at 2 × 10⁵ PFU, reflecting its historical use in smallpox vaccination programs (36). Serum samples were collected on day 14 (post-primary immunization) and day 28 (post-booster), and enzyme-linked immunosorbent assay (ELISA) was performed using inactivated VACV virions as coating antigen to assess total orthopoxvirus-specific IgG levels. Total anti-VACV IgG titers were detected in all groups on day 14 (Fig. 5B). The VTT group exhibited the highest titer (mean: 3.73 × 10⁴), significantly exceeding the titers in both NTV and NTV-ΔF1L-C7L groups (*P* < 0.05). Among intramuscular vaccines, NTV-ΔF1L-C7L induced higher IgG levels than NTV at all doses. Following booster immunization, IgG titers increased substantially in both NTV-based groups. At the 10⁵ PFU dose, the NTV-ΔF1L-C7L group achieved a mean titer of 1.52 × 10⁴ at day 28, which was significantly higher than that in the corresponding NTV group (4.2 × 10³; *P* < 0.0001) and even surpassed the single-dose 10⁷ PFU NTV-ΔF1L-C7L titer (1.07 × 10⁴) at day 14. These results indicate that booster vaccination effectively compensates for a low primary dose.

**Fig 6:**
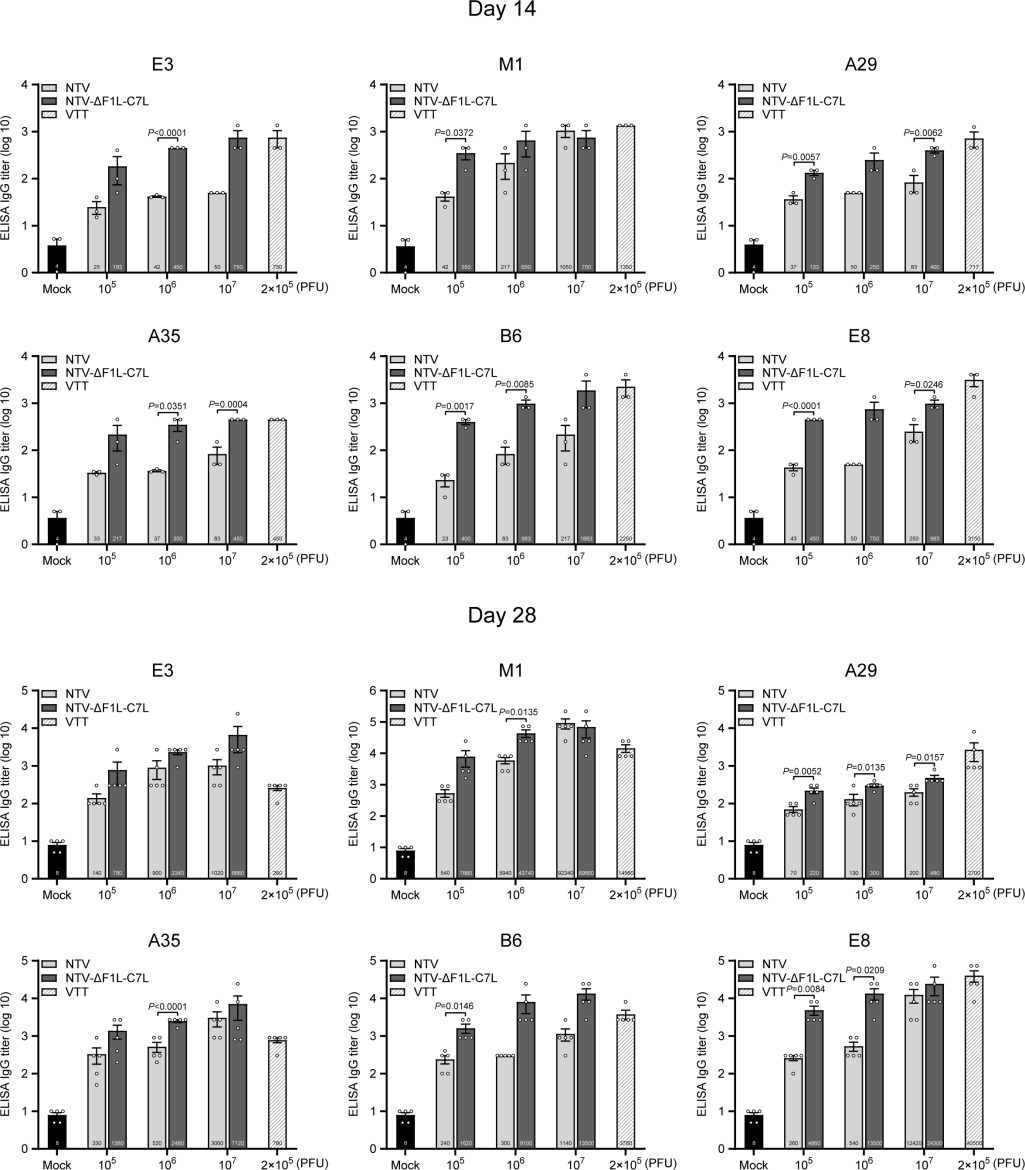
Anti-MPXV antigen-specific IgG responses. IgG titers against six MPXV early and late proteins in mouse serum at 14 and 28 days after immunization, determined by ELISA. Data are presented as mean ± SEM. Statistical significance was assessed by one-way ANOVA with multiple comparisons; *P*-values for comparisons between NTV-ΔF1L-C7L and NTV, and between NTV-ΔF1L-C7L and VTT, are shown above the bars where applicable.

In all vaccine groups, anti-orthopoxvirus-specific IgG responses against all six MPXV core proteins (E3, A29, M1, E8, B6, and A35) were detected (Fig. 6). Except for A29, IgG titers against the remaining antigens—including the early protein E3, intracellular mature virion (IMV)-associated proteins (M1, E8), and EEV-associated proteins (B6, A35)—followed the same trend as total anti-VACV IgG: NTV-ΔF1L-C7L induced higher antibody titers than NTV, particularly at lower doses (10⁵–10⁶ PFU), with further enhancement after booster immunization. Anti-A29 IgG levels were consistently lower than those against other antigens across all groups.

### NTV-ΔF1L-C7L induces potent neutralizing antibody responses against VACV and MPXV

**Fig 7:**
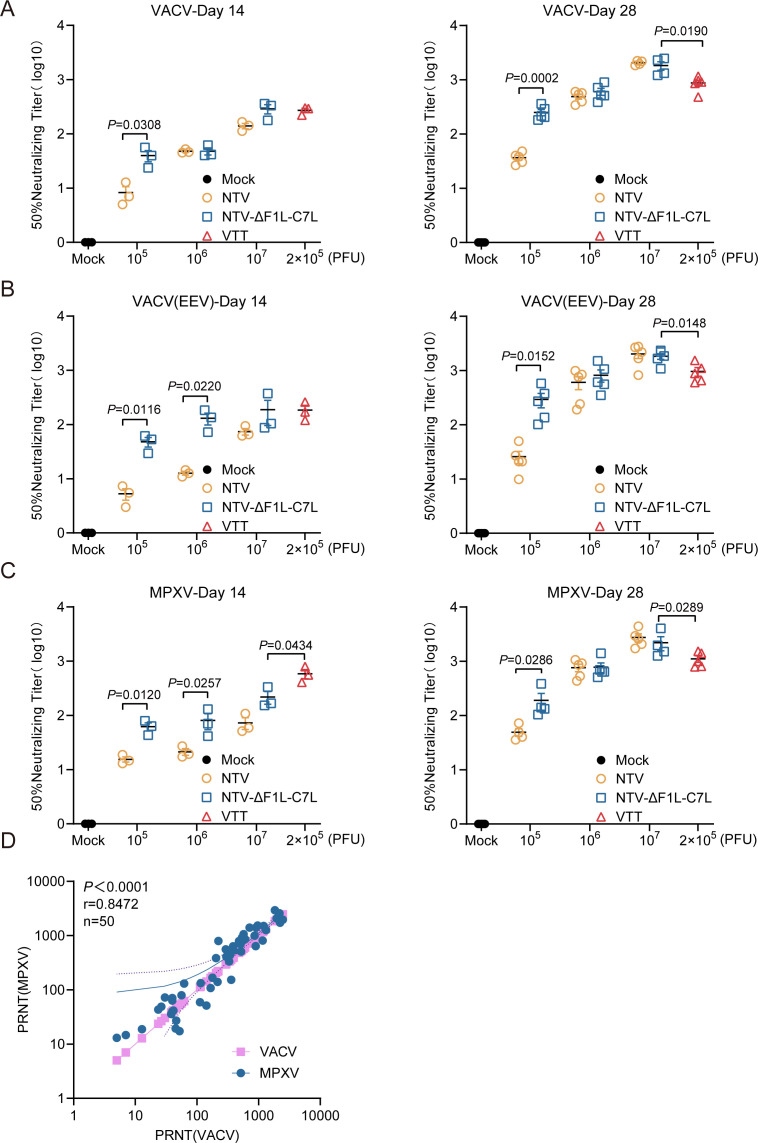
Neutralizing antibody responses and cross-neutralization correlation. (A–C) Neutralizing antibody titers against VTT (**A**), VTT extracellular enveloped virus (**B**), and MPXV (**C**) in mouse serum at 14 and 28 days after primary immunization, measured by PRNT. (**D**) Correlation between VTT-NT₅₀ and MPXV-NT₅₀ based on PRNT assays. Regression models were fitted to log-transformed titers; the *r* and *P* values are shown, and the 95% confidence interval is represented by dotted lines. All experiments were performed independently. Data are presented as mean ± SEM. Statistical comparisons were performed using one-way ANOVA with multiple comparisons test, with *P*-values for NTV-ΔF1L-C7L vs NTV and NTV-ΔF1L-C7L vs VTT indicated directly above the relevant bars.

Neutralizing antibody (nAb) titers against VACV were assessed via plaque reduction neutralization test (PRNT) on days 14 and 28 post-immunization (Fig. 7A). Following primary immunization, all vaccinated groups exhibited detectable orthopoxvirus-specific nAbs. The VTT group induced higher geometric mean titers (GMTs) than the NTV-based groups. Among the latter, NTV-ΔF1L-C7L elicited significantly higher nAb titers than NTV at the 10⁵ PFU dose (*P* = 0.0308; approximately fivefold higher based on GMTs), although this difference diminished at higher doses. At 10⁷ PFU, NTV-ΔF1L-C7L reached GMT levels comparable to those of VTT. After booster immunization, nAb titers increased significantly in all groups. VTT showed more than a fourfold increase in GMT from day 14 to day 28. In the NTV series, a clear dose-dependent response was observed: at 10⁵ PFU, NTV-ΔF1L-C7L induced nAb titers significantly higher than NTV (*P* = 0.0002; approximately sevenfold higher based on GMTs), and at 10⁶ PFU, titers approached those of VTT. These trends were consistent with total IgG measurements. To further assess neutralizing activity against extracellular virions, we measured nAb titers against VACV extracellular enveloped virus (EEV) (Fig. 7B). The results followed a pattern similar to that observed for whole-virus neutralization: NTV-ΔF1L-C7L induced higher anti-EEV nAb titers than NTV across all doses, with a more pronounced difference at the 10⁵ PFU dose. After booster immunization, the 10⁶ PFU NTV-ΔF1L-C7L group achieved neutralizing titers comparable to those in the VTT group. These results indicate that antibodies elicited by this vaccine effectively neutralized both IMV and EEV forms of VACV.

Cross-neutralization analysis against MPXV revealed a similar trend (Fig. 7C). nAb titers followed the same group ranking (VTT ≥ NTV-ΔF1L-C7L > NTV) and dose-dependent responses as those observed for VACV. Regression analysis of PRNT data confirmed a strong positive correlation between VACV and MPXV nAb titers (*r* = 0.8472, *P* < 0.0001, *n* = 50), supporting the use of VACV-based neutralization as a surrogate for MPXV assessment (Fig. 7D).

### NTV-ΔF1L-C7L induces robust Th1-biased cellular immunity

**Fig 8:**
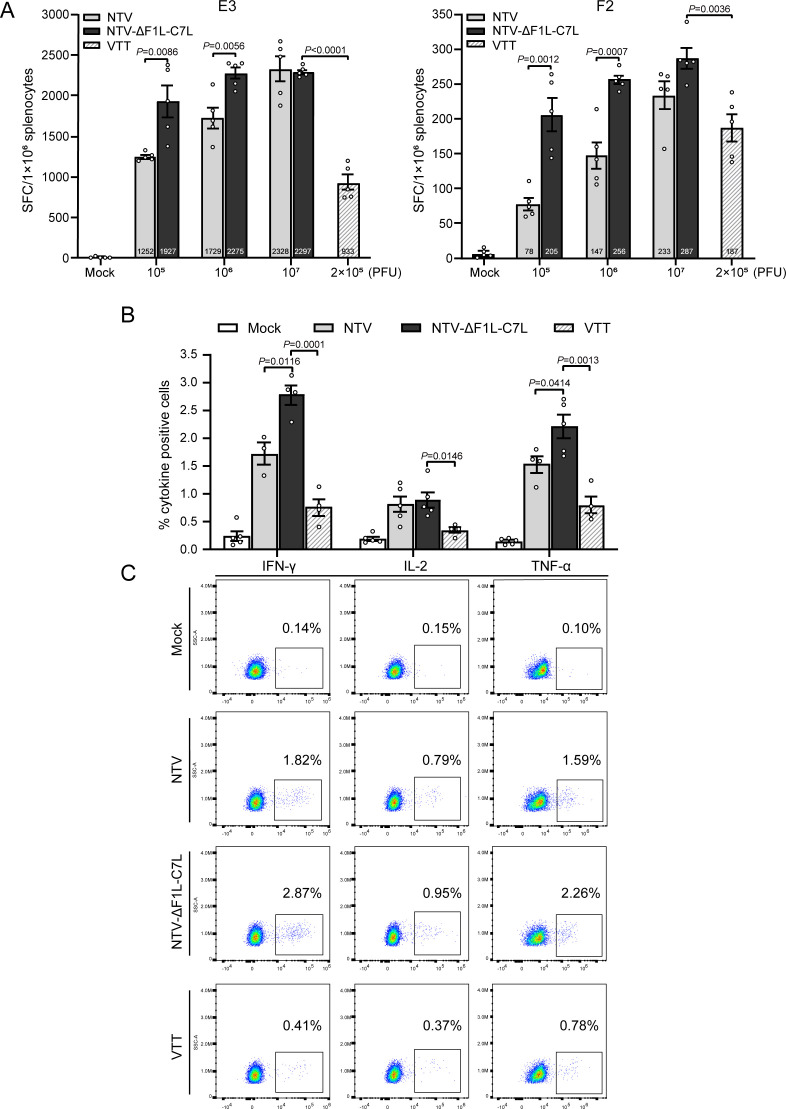
NTV-ΔF1L-C7L induces cellular immune response. (**A**) Mouse splenocytes (*n* = 5) were collected 28 days after immunization with NTV, NTV-ΔF1L-C7L, and VTT vaccines, and the number of positive cells secreting IFN-γ by T cells from immunized mice stimulated with E3 (140–148, VGPSNSPTF) and F2 (26–34, SPYAAGYDL) peptides was determined using ELISpot . (**B and C**) Mouse splenocytes were processed and stimulated with E3 (140–148, VGPSNSPTF) peptide 28 days after immunization at a dose of 10⁵ PFU for each group, and the percentages of CD8+ splenocytes positive for IFN-γ, TNF-α, and IL-2 were determined using flow cytometry. Corresponding to Fig. 5B, representative cytokine expression levels of mice in each group are shown to intuitively compare the differences between groups. The above experiments were performed independently. Data are reported as mean ± SEM. Statistical significance was calculated using one-way ANOVA with multiple comparisons test. To clearly indicate specific differences, *P*-values for comparisons between NTV-ΔF1L-C7L and NTV, as well as between NTV-ΔF1L-C7L and VTT, are shown directly above the corresponding bars.

Cellular immunity is critical for effective vaccine-mediated protection. T -cell responses elicited by NTV-ΔF1L-C7L were evaluated by isolating splenocytes from immunized mice on day 28 and analyzing them using enzyme-linked immunospot (ELISpot) assay and intracellular cytokine staining (ICS). As shown in Fig. 8A, ELISpot analysis revealed that stimulation with the E3 peptide induced considerably more IFN-γ^+^ spot-forming cells (SFCs) than stimulation with the F2 peptide (approximately 10-fold), although both peptides exhibited a similar dose-response profile across all vaccine groups. At immunization doses of 10⁵–10⁶ PFU, the NTV-ΔF1L-C7L group induced a significantly stronger antigen-specific IFN-γ response than that of the NTV group (*P* < 0.01). However, at the 10⁷ PFU dose, no significant difference was observed between the two groups, and the number of IFN-γ^+^ SFCs plateaued, suggesting potential immune tolerance. The VTT group exhibited a weaker IFN-γ response than the NTV-ΔF1L-C7L group at the dose of 10⁵ PFU. CD8^+^ T -cell responses were further characterized by assessing cytokine profiles of E3-stimulated splenocytes via ICS. As shown in Fig. 8B, at a 10⁵ PFU dose, the CD8^+^ T cells in the NTV-ΔF1L-C7L group produced markedly higher levels of IFN-γ and TNF-α than those in the NTV group (*P* < 0.05), whereas IL-2 production was relatively low. The VTT group exhibited markedly lower secretion of all measured cytokines (Fig. 8C). IL-4 levels were comparable across all groups and did not differ from those of the mock group (data not shown), indicating that all vaccine candidates predominantly elicited a Th1-type immune response.

### NTV-ΔF1L-C7L protects mice from lethal VACV Western Reserve challenge

**Fig 9:**
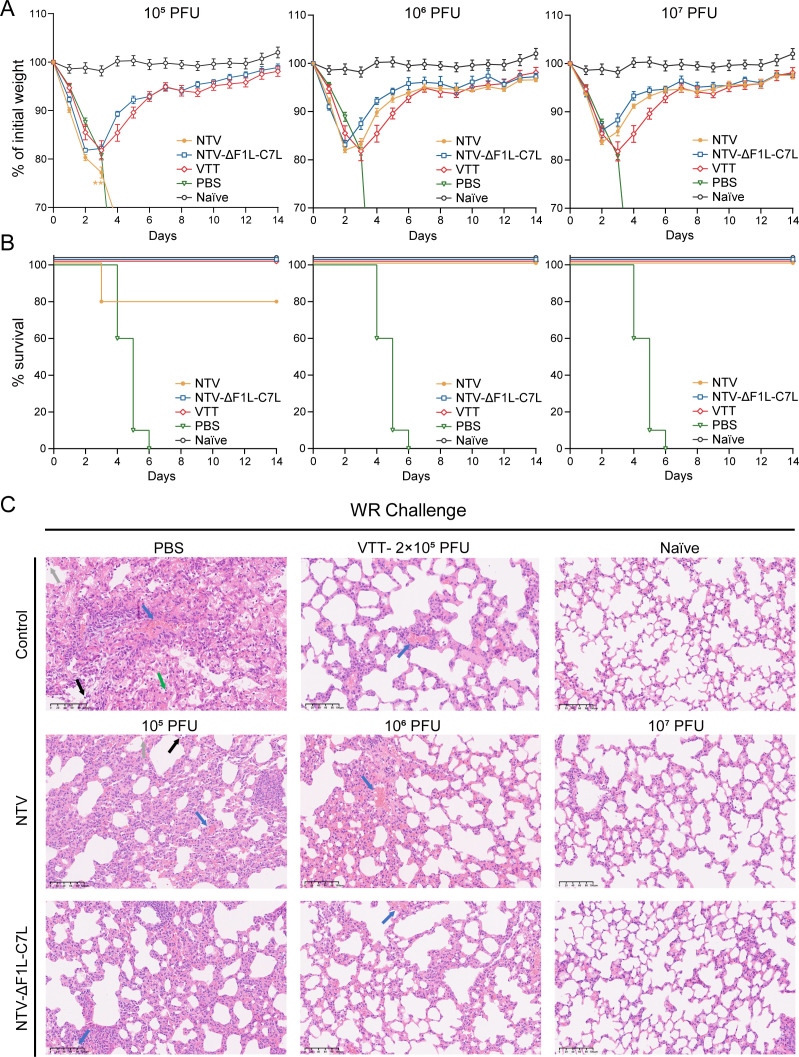
NTV-ΔF1L-C7L protects BALB/c mice from lethal VACV WR challenges. Groups of 6-week-old female BALB/c mice (*n* = 13) were immunized intramuscularly with 10⁵–10⁷ PFU of NTV and NTV-ΔF1L-C7L two times at a 2-week interval or once with 2 × 10⁵ PFU of VTT via tail scarification. The negative control group (*n* = 26) was intramuscularly injected with PBS as a mock vaccine. After 6 weeks, mice were intranasally challenged with 10⁷ PFU (25× LD₅₀) of VACV WR. The negative control group was divided into two groups (*n* = 13), one of which was the PBS group that received the WR challenge and the other was the naïve group that received the PBS mock challenge. The mice were monitored for weight loss and mortality for 14 consecutive days. (**A**) Changes in body weight of mice over 14 days after VACV WR challenge, with the number of deceased animals noted on the corresponding day of the curve with an asterisk (*). (**B**) Survival curve of mice over 14 days after VACV WR challenge, with weight loss exceeding 25% considered as death, and the animals were euthanized. (**C**) Hematoxylin and eosin staining of lung sections from vaccinated and control animals (*n* = 3) on day 5 after challenge. Gray arrows indicate necrosis and shedding of bronchial mucosal epithelial cells, leading to bronchial structural damage. The bronchial lumen contained almost no necrotic cell debris and eosinophilic secretions. The alveolar spaces contained a few macrophages (green arrows) and lymphocytes (black arrows). Blue arrows indicate interstitial vascular congestion. Scale bar, 100 μm.

Challenge protection is a key indicator for evaluating the overall efficacy of vaccine candidates. In this study, protective efficacy was evaluated by intranasally challenging immunized mice (*n* = 13 per group) with a high dose of virulent VACV Western Reserve (WR) (10⁷ PFU; 25× LD₅₀) on day 42 post-primary immunization. As shown in Fig. 9A and B, all groups exhibited varying weight loss at 1 dpi. The PBS control group experienced rapid weight decline, reaching 100 % mortality by 6 dpi. The VTT group exhibited typical protective characteristics, with maximum weight loss restricted to ~17% at 3 dpi and the survival rate reaching 100%. In contrast, NTV-based vaccines demonstrated a dose-dependent protective effect. At 10⁵ PFU, the NTV group exhibited a maximum weight loss of 22% and 80% survival, with two mice exceeding the 25% loss threshold and requiring euthanasia. In comparison, the NTV-ΔF1L-C7L at the same dose resulted in ~17% wt loss, comparable to that of VTT, and showed slightly faster recovery. At higher doses (10⁶–10⁷ PFU), differences between NTV and NTV-ΔF1L-C7L further diminished, with both achieving near-complete protection.

Histopathological analysis at 5 dpi (Fig. 9C) confirmed the clinical protection data. PBS-treated mice exhibited severe pulmonary lesions—widespread alveolar necrosis, interstitial lymphocytic infiltration, and thickened alveolar septa—with a mean pathology score of 4.0 (Table 2). In contrast, all vaccinated groups showed markedly reduced pathology. NTV-ΔF1L-C7L induced minimal lung damage: at 10⁵ PFU, only focal congestion was observed, with a pathology score of 1.67—comparable to VTT (1.67) and significantly lower than NTV, which exhibited moderate inflammation with focal cellular infiltration (score = 2.33; *P* < 0.05). At 10⁶ PFU, NTV-ΔF1L-C7L maintained superior protection with minimal pathological changes (score = 1.33), whereas NTV showed moderate but reduced inflammation compared with the lower dose, characterized by scattered cellular infiltration (score = 2.0; *P* < 0.05). At the highest dose (10⁷ PFU), both vaccines preserved near-normal lung architecture, reflected by equally low scores (0.67), indistinguishable from naïve controls.

**TABLE 2:** Mouse lung pathology scoring table after VACV WR challenge

Group	Immunization dose (PFU)	Pathology score^*a*^
Naïve		0
PBS		4
VTT	2 × 10^5^	1.67
NTV	10^5^	2.33
	10^6^	2
	10^7^	0.67
NTV-ΔF1L-C7L	10^5^	1.67
	10^6^	1.33
	10^7^	0.67

^*a*^ The pathology scoring system for mouse lung tissue ranges from 0 to 5, with higher scores indicating more severe changes: 0 (no significant pathology), 1 (mild pathology with slight thickening and sparse infiltration), 2 (moderate pathology with significant thickening and increased infiltration), 3–4 (severe pathology with marked thickening and extensive infiltration), and 5 (very severe pathology with extensive exudation and hemorrhage). The pathology score for each group represents the mean score of three mice.

These results demonstrate that NTV-ΔF1L-C7L confers enhanced protection compared to its parental strain, particularly at lower immunization doses, and that high-dose immunization effectively prevents pulmonary pathology caused by virulent VACV WR.

### Evaluation of NTV-ΔF1L-C7L immunogenicity at lower doses

**Fig 10:**
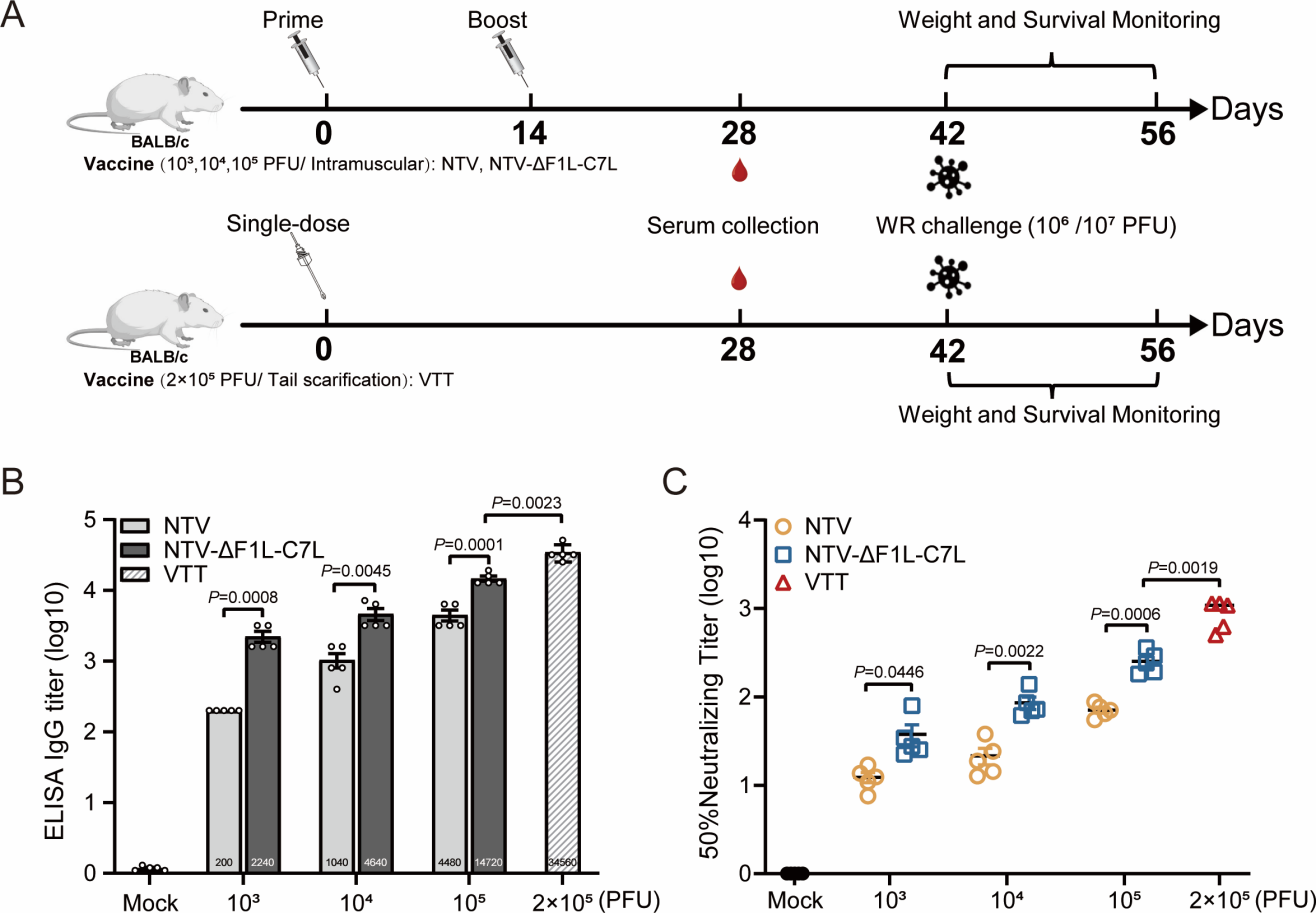
Immunization schedule and humoral immune responses at reduced immunization doses. (**A**) Immunization schedule. Female 6-week-old BALB/c mice were randomly divided into groups (*n* = 5) and immunized intramuscularly with 10³–10⁵ PFU of NTV or NTV-ΔF1L-C7L twice at a 2-week interval, or once with 2 × 10⁵ PFU of VTT via tail scarification. The negative control group (*n* = 10) received PBS intramuscularly. Serum samples were collected from five mice per group at 28 days after primary immunization for humoral immune analysis. (**B**) Anti-VACV IgG titers in mouse serum at 28 days after primary immunization, measured by ELISA. (**C**) Neutralizing antibody titers against VTT and MPXV in mouse serum at 28 days after primary immunization, determined by PRNT. Data are presented as mean ± SEM. Statistical significance was assessed using one-way ANOVA with multiple comparisons. *P*-values for comparisons between NTV-ΔF1L-C7L and NTV, and between NTV-ΔF1L-C7L and VTT, are shown directly above the corresponding bars.

The immunization-dose relationship was further refined by immunizing additional cohorts with reduced doses (10³, 10^4^, and 10⁵ PFU) to determine the minimum effective immunization dose (Fig. 10A).

**Fig 11:**
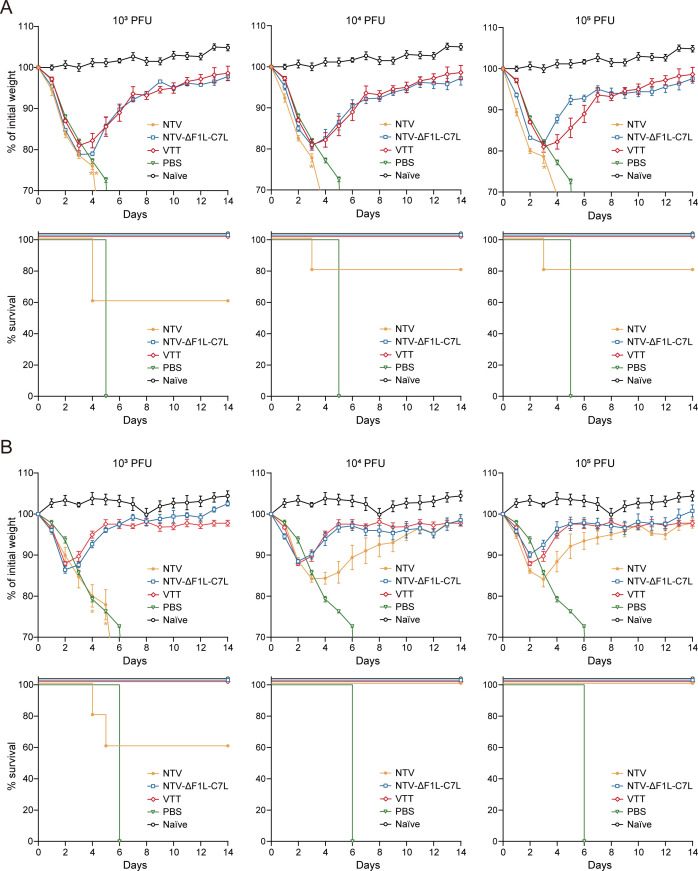
Protective efficacy of NTV-ΔF1L-C7L at reduced immunization doses against lethal VACV WR challenge. Forty-two days after primary immunization, all immunized mice and PBS controls were challenged with VACV WR, and body weight changes and survival were monitored for 14 consecutive days. Deceased animals are indicated by an asterisk (*) on the corresponding day of the weight curve; mice losing more than 25% of their body weight were considered dead and euthanized. (**A**) Body weight changes and survival curves of mice challenged with 10⁷ PFU (25× LD₅₀) of VACV WR. (**B**) Body weight changes and survival curves of mice challenged with 10⁶ PFU (2.5× LD₅₀) of VACV WR. Data are reported as mean ± SEM. Statistical significance was assessed using one-way ANOVA with multiple comparisons.

Mouse sera collected on day 28 were analyzed for antibody responses. As shown in Fig. 10B, the IgG levels in the 10⁵ PFU group were consistent with earlier results (Fig. 5B), indicating the reproducibility of the experiment. Across the 10³–10⁵ PFU range, NTV-ΔF1L-C7L induced significantly higher antibody titers than NTV at all doses (*P* < 0.01), with mean IgG levels over fourfold greater. nAb titers (Fig. 10C) were consistent with the IgG response, confirming the superior humoral immunogenicity of NTV-ΔF1L-C7L at lower doses. Protective efficacy was assessed by challenging immunized mice with 10⁷ PFU of VACV WR on day 42, followed by monitoring weight change and survival over 14 days. As shown in Fig. 11A, NTV-ΔF1L-C7L conferred robust protection, even at low doses. The 10⁴ PFU group exhibited a weight loss pattern similar to that of the VTT. The 10³ PFU group showed a maximum weight loss of 21%; however, it achieved 100% survival, significantly better than the 60% survival rate observed in the NTV group at the same dose (*P* < 0.05).

However, under this stringent 10⁷ PFU challenge condition, the substantial body weight fluctuations observed in vaccinated mice precluded an accurate assessment of the protective efficacy of NTV-ΔF1L-C7L, particularly at lower immunization doses. To better reflect a viral load close to that of a natural infection and obtain a clearer evaluation of vaccine-induced protection, we reduced the challenge dose 10-fold to 10⁶ PFU VACV WR. Mice were immunized with two doses (10³, 10⁴, and 10⁵ PFU) of either NTV or NTV-ΔF1L-C7L and then challenged with 10⁶ PFU VACV WR. As shown in Fig. 11B and Table S1, at the reduced challenge dose, both NTV-ΔF1L-C7L- and VTT-immunized groups exhibited markedly attenuated body weight loss (approximately 12% at peak) compared with the 10⁷ PFU challenge. Vaccination with NTV-ΔF1L-C7L induced a robust, dose-dependent reduction in clinical severity. Mice immunized with NTV-ΔF1L-C7L, even at the lowest dose (10³ PFU), maintained a relatively healthy physiological status, with a maximum mean clinical score of 7.0 during the acute phase (days 1–3 post-challenge) (Table S1). In contrast, the NTV -immunized group showed significantly exacerbated morbidity under the same challenge conditions. Mice receiving the 10³ PFU dose of NTV exhibited severe clinical symptoms, with mean scores ranging from 15.2 to 17.2 during the observation period, indicative of a severe state. Moreover, this group displayed a drastic decline in survival, with only 60% of mice surviving at the 10³ PFU dose. These results underscore the superior safety profile and protective capacity of the NTV-ΔF1L-C7L construct, particularly at suboptimal immunization doses.

### Evaluation of NTV-ΔF1L-C7L protective efficacy following single-dose immunization

**Fig 12:**
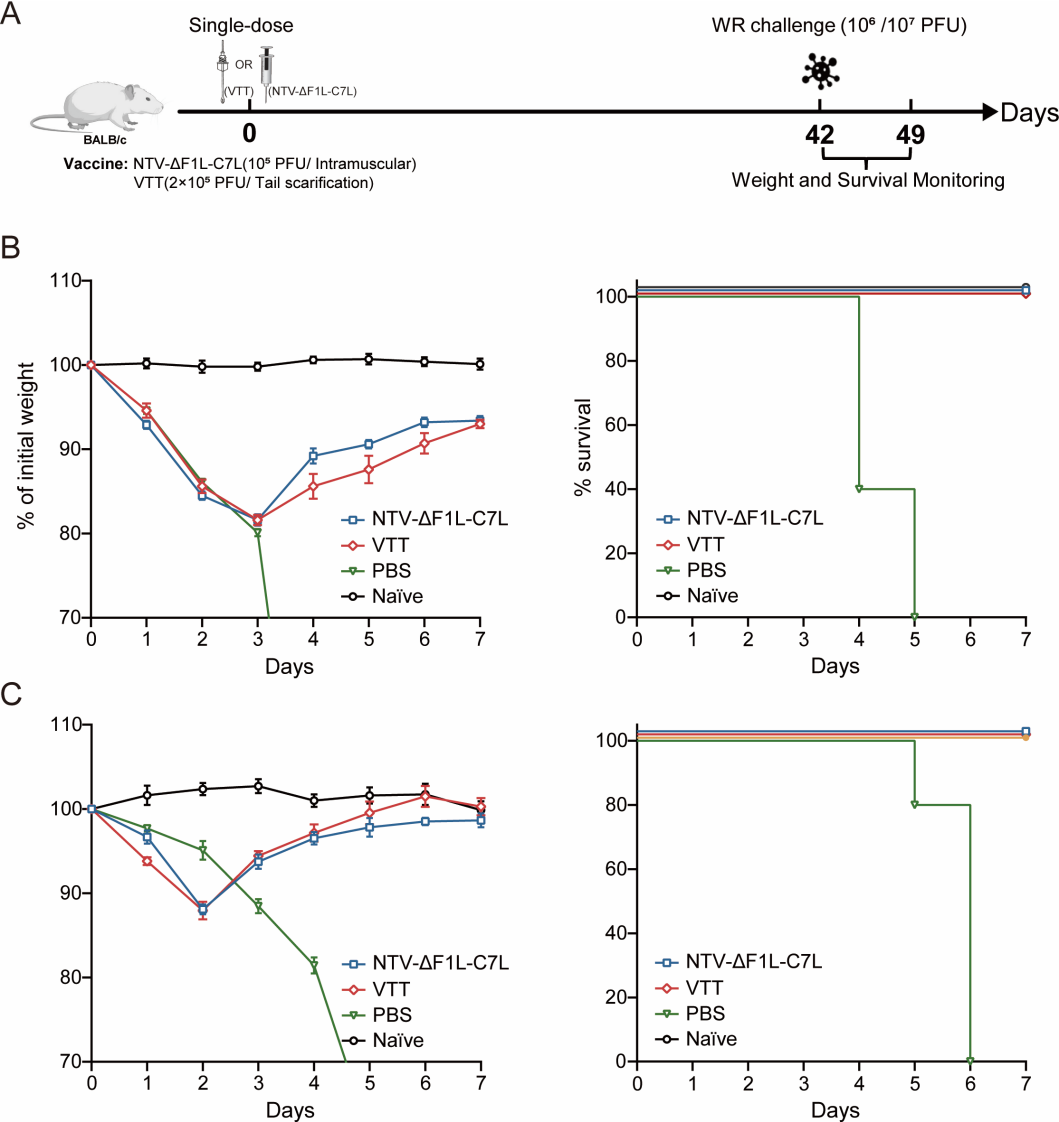
Protection against lethal challenge following single-dose immunization with NTV-ΔF1L-C7L. (**A**) Immunization schedule of the vaccines. Female 6-week-old BALB/c mice were randomly divided into groups (*n* = 5). Mice were immunized intramuscularly with 10⁵ PFU of NTV-ΔF1L-C7L or 2 × 10⁵ PFU of VTT by tail scarification. The negative control group (*n* = 10) was intramuscularly injected with PBS as a mock vaccine. At 42 days post-immunization, all groups were challenged with 10⁶ PFU (2.5× LD₅₀) or 10⁷ PFU (25× LD₅₀) of VACV WR. (**B**) Body weight changes and survival curves of mice 7 days after 10⁷ PFU (25× LD₅₀) VACV WR challenge. Mice that lost more than 25% of their body weight were considered dead and were euthanized. (**C**) Body weight changes and survival curves of mice 7 days after 10⁶ PFU (2.5× LD₅₀) VACV WR challenge. Mice that lost more than 25% of their body weight were considered dead and were euthanized.

To further evaluate the protective efficacy of single-dose immunization with NTV-ΔF1L-C7L, we measured the body weight changes and survival of mice immunized with 10⁵ PFU of NTV-ΔF1L-C7L and challenged with 10⁷ PFU of VACV WR for 7 days (Fig. 12A). As shown in Fig. 12B, the situation of the VTT group after the challenge was similar to the aforementioned results (Fig. 9A and B and Fig. 11), which confirmed the stability of the challenge experiment. In comparison, the NTV-ΔF1L-C7L group showed a similar trend of body weight loss to the VTT group, but the recovery of body weight was faster. Moreover, it provides complete survival protection. Its protective efficacy against challenge was close to that of the booster immunization group at the same dose (Fig. 11A).

To better discern the protective efficacy and group differences, we additionally performed a single-dose immunization experiment with a reduced challenge dose of 10⁶ PFU VACV WR. Under this condition, mice immunized with a single dose (10⁵ PFU) of either VTT or NTV-ΔF1L-C7L exhibited a maximum body weight loss of approximately 12% (Fig. 12C). Clinical scoring (Table S2) further confirmed comparable protection between the two groups, which showed no significant differences in clinical scores and achieved 100% survival. These data demonstrate that under a 10⁶ PFU challenge, single-dose immunization with NTV-ΔF1L-C7L provides protective efficacy equivalent to that of VTT.

## DISCUSSION

The recent mpox outbreak has exposed critical limitations in the production of currently approved vaccines. Existing platforms predominantly rely on primary cell cultures, such as the JYNNEOS vaccine produced in CEF cells (37) and the recently WHO Emergency Use Listing LC16m8 vaccine, which requires primary rabbit kidney cells (38). These primary cell-based systems are operationally complex, subject to stringent quality controls, and hampered by high batch-to-batch variability and limited scalability. Consequently, they incur elevated production costs and contribute to an insufficient global vaccine supply (39). This scenario highlights the urgent need for alternative vaccine production platforms using continuous cell lines that enable simplified manufacturing processes and cost-effective scalability. Previous studies have comprehensively compared the host range characteristics of VTT and NTV (7, 21). VTT efficiently replicates in multiple cell lines commonly used for vaccine production, including Vero, MRC-5, and MDCK cells, whereas NTV effectively proliferates primarily in CEF cells. Given that the recombinant virus in this study was derived from NTV, assessing alterations in its replication capacity was a primary focus. Consistent with vaccine vector design principles, replication was evaluated in CEF, Vero, and MRC-5 cells. Compared with NTV, NTV-ΔF1L-C7L demonstrated a significant enhancement in viral yield over a 72 h culture period, with viral titers in MRC-5 cells increasing up to 680-fold relative to those of the parental strain.

This marked expansion in host range and replication efficiency is likely attributable to restoration of the C7L gene. C7L is a highly conserved host-range gene that antagonizes the host restriction factor, SAMD9. Deletion of C7L impairs late viral gene expression, and a similar dependence on SAMD9 has been observed for NTV (21, 40–46). Western blot analysis using MPXV-specific antibodies demonstrated that NTV (engineered to lack C7L) produced barely detectable levels of VACV late proteins cross-reactive with MPXV antibodies, whereas early protein E3 was expressed normally. In contrast, NTV-ΔF1L-C7L fully restored the expression of these late proteins to levels comparable to those of VTT. This result is consistent with the notion that C7L complementation alleviates host-mediated suppression of late gene expression and aligns with previous studies indicating that replication of K1L/C7L-deficient viruses may be hindered at the transition from intermediate to late gene expression (42, 47). Vero cells are permissive for C7L-deficient viruses due to defective type I interferon signaling and consequently low SAMD9 expression, which explains the permissive phenotype observed in earlier reports.

In summary, reintroduction of C7L into the C7L-deficient NTV backbone effectively overcomes the host defense imposed by the SAMD9/SAMD9L family of restriction factors, thereby restoring efficient viral replication in multiple continuous cell lines—including HeLa, MRC-5, and Vero. Advancement of NTV-ΔF1L-C7L as an mpox vaccine candidate could help mitigate the current supply and demand disparity associated with limited production capacity. These constraints have prompted dose-sparing regimens, administering a single dose instead of two, and production profiles of NTV-ΔF1L-C7L thus present a more practical alternative to meet global vaccination needs (48, 49).

In this study, we systematically evaluated the immunological properties of NTV-ΔF1L-C7L in BALB/c mice. Humoral immune analysis demonstrated that this vaccine effectively induced VACV- and MPXV-specific IgG antibodies. Moreover, it elicited robust responses against all six core MPXV antigens, including the early protein E3 and the late proteins M1, A29, A35, B6, and E8. The antibody titers against each antigen were significantly higher than those induced by the parental NTV strain, particularly at the low dose of 10⁵ PFU. Consistent with this result, NTV-ΔF1L-C7L also elicited markedly enhanced nAb responses, exhibiting superior neutralization activity against whole VACV virions and EEVs. This enhanced EEV neutralization correlates strongly with elevated antibody levels against EEV-associated antigens, such as B6 and A35. Cellular immune assessment using ELISpot and ICS further confirmed that NTV-ΔF1L-C7L significantly augmented Th1-type responses. This was evidenced by greater frequencies of antigen-specific CD8^+^ T cells producing IFN-γ, TNF-α, and IL-2. These responses even surpassed those induced by the conventional VTT at the same dose.

These immunological advantages are attributed to the restoration of viral antigen expression. As shown using western blotting, C7L complementation enabled the production of late viral antigens by antagonizing SAMD9/SAMD9L (47), and this antagonism is conserved across species (50), suggesting that late antigen production *in vitro* may translate to immunogenicity *in vivo* .

Deletion of F1L may further influence the immune response. Infection with NTV-ΔF1L-C7L induced cleavage of caspase-9, caspase-8, caspase-3, and PARP, indicating that loss of F1L function relieves suppression of the intrinsic apoptotic pathway. The concurrent activation of caspase-8 also suggests engagement of the extrinsic apoptotic pathway, potentially amplifying the apoptotic signal. This apoptotic process has been associated with immunogenic cell death, and the resulting apoptotic bodies could serve as a source of antigen for dendritic cell uptake and cross-presentation, thereby contributing to the enhanced CD8^+^ T -cell responses observed in our study (34). Given the evolutionary conservation of this core apoptotic pathway, the pro-immunogenic effects of F1L deletion observed in human cells may be relevant to the enhanced CD8^+^ T -cell responses observed in BALB/c mice (34). Therefore, C7L and F1L may play complementary roles: C7L enables sufficient production of late viral antigens, whereas F1L deletion promotes their immunogenic presentation via apoptosis. Nevertheless, direct causal evidence linking F1L deletion-induced apoptosis to the observed immunogenicity *in vivo* remains to be established, and further studies (e.g., using caspase inhibitors or genetic manipulations) are warranted.

In this context, this coordinated humoral and cellular immunity is essential for achieving comprehensive protection. Historically, the role of virus-specific antibodies has been emphasized in protection against lethal orthopoxvirus infections (51, 52). However, the results of the present study indicate that cellular immunity, particularly the CD8^+^ T -cell response, can compensate for suboptimal humoral immunity. This supports the emerging consensus that coordinated humoral and cellular immunity is essential for effective anti-VACV protection (53–56). Vaccination route analysis revealed that VTT administered via scarification induced weaker cellular immunity, consistent with reports that ACAM2000, when delivered by scarification, elicits minimal Th1-type cytokine responses (57). In contrast, intradermally administered Dryvax has been shown to induce strong cellular responses (58), suggesting that scarification is a limiting factor in the cellular immunogenicity of replication-competent VACV. In parallel, immunogenicity comparisons across administration routes (scarification vs intramuscular injection) for MVA showed no significant differences (58), highlighting that the influence of vaccination route differs between replication-competent and non-replicating VACV strains. Thus, for replication-competent VACV vaccines administered via scarification, virus-specific antibody levels appear to play a more decisive role in protective efficacy.

A systematic evaluation of the immunization dose effects of NTV-ΔF1L-C7L revealed that higher dose ranges (10⁵–10⁷ PFU) masked differences in immunogenicity between vaccine strains, consistent with the dose saturation effect previously reported by Moss et al. (57). Although initially attributed to the small sample size (*n* = 4), expansion of the cohort (*n* = 10) confirmed that a dose of 10⁵ PFU failed to distinguish intergroup differences. The present study results show that, at high doses, vaccines with robust immunogenicity do not elicit further increases in protective efficacy, whereas those with weaker immunogenicity exhibit more pronounced dose-dependent effects. These findings suggest that the 10⁵–10⁷ PFU range may exceed the optimal evaluation window in mice, hindering accurate comparison of immune responses among vaccine candidates. When the immunization dose was adjusted to 10³–10⁵ PFU, the resulting data were more distinguishable. Within this range, NTV-ΔF1L-C7L exhibited a clear dose–response relationship, and antibody titers were significantly higher than those induced by NTV at all tested doses. NTV-ΔF1L-C7L conferred 100% protection against all challenges, whereas NTV achieved only 60–80% protection.

However, under the stringent 10⁷ PFU challenge, substantial body weight fluctuations were observed across all immunized groups, complicating precise assessment of the protective capacity of NTV-ΔF1L-C7L, particularly at lower immunization doses. Considering that this dose possibly exceeds the viral load encountered during natural infection, we reduced the challenge dose 10-fold to 10⁶ PFU (2.5× LD₅₀). Under these more physiologically relevant conditions, the differences in protection between NTV-ΔF1L-C7L and NTV became markedly more distinct, and the superior efficacy of NTV-ΔF1L-C7L was clearly demonstrated even at the lowest immunization dose. Collectively, this dose optimization study establishes 10³ PFU as the minimum effective protective dose of NTV-ΔF1L-C7L, providing a critical reference point for subsequent vaccine dose selection. Single-dose immunization challenge results showed that the recombinant virus, at a dose twofold lower than VTT, conferred protective efficacy comparable to that of VTT, further confirming the protective potential of the recombinant virus. In contrast, a recently published study on a genetically modified attenuated strain derived from VTT did not even compare its protective efficacy with VTT in the challenge model, but only with the non-replicating vaccinia virus MVA (59).

Compared with existing platforms, such as mRNA vaccines that elicit robust nAb and T -cell responses (60, 61), the challenge of protection they confer remains comparable to that of attenuated live vaccines, such as LC16m8 and MVA-BN (62, 63). Attenuated live vaccines simulate natural infection, thereby inducing both humoral and cellular immunity, with the added advantage of long-term protection from a single dose (64). This observation aligns with the hypothesis of Liu et al. that replication-competent VACV enhances immune responses by prolonging antigen exposure (65). Supporting this hypothesis, Li et al. demonstrated that specific IgG antibodies induced by VTT persisted for over 42 years in historically vaccinated individuals, highlighting the potential for long-term immunity conferred by replication-competent VACV-based vaccines (66). Overall, the findings that the attenuated live vaccine candidate NTV-ΔF1L-C7L exhibits strong immunogenicity, complete immune protection efficacy, and replication competence suggest its potential for long-term immune protection.

Currently, various strategies have been adopted to use the limited vaccine resources more efficiently to protect more people. The US Food and Drug Administration approved the intradermal administration of the JYNNEOS vaccine on 9 August 2022, with a dosage that was only 20% of the conventional subcutaneous injection (67). Meanwhile, in cases of extreme shortage, single-dose immunization has also been proven to provide acceptable protective efficacy among high-risk populations (68). Preliminary data from our study indicated that the recombinant VACV NTV-ΔF1L-C7L can induce an effective immune response at an extremely low dose. Therefore, its single-dose protective potential was further assessed. The results showed that, at a dose of only half of the traditional VTT immunization dose (2 × 10⁵ PFU), NTV-ΔF1L-C7L could provide equivalent and even faster weight recovery kinetics against a lethal WR challenge of 10^7^ PFU compared to VTT, highlighting its potential advantage in a single-dose, low-dose regimen. In comparison, the VTT recombinant strain with F4L, TK, and B2R triple-gene deletion, as reported by Guo et al. (69), although possessing good safety and immunogenicity, achieved complete protection against a VTT challenge of 2.5 × 10⁵ PFU only after a single-dose immunization of 10⁵ PFU. However, its challenge model was relatively mild. The NTV-ΔF1L-C7L in our study demonstrated resistance to a more severe lethal challenge, suggesting a potentially higher protective threshold. Nonetheless, the protective efficacy of NTV-ΔF1L-C7L against MPXV requires systematic evaluation.

In addition to expanding the host range to enhance viral yield and immunogenicity, the recombinant virus is expected to exhibit improved safety characteristics, a feature likely attributable to the deletion of F1L, a well-established orthopoxvirus virulence factor (as discussed above). In our study, infection with NTV-ΔF1L-C7L markedly enhanced cleavage of caspase-9 and PARP, suggesting that loss of F1L function may relieve inhibition of the intrinsic apoptotic pathway. Consistent with this molecular phenotype, the virus displayed profound attenuation in BALB/c mice: even at an intranasal dose of 10⁷ PFU—10-fold higher than the lethal dose of VTT (10⁶ PFU, which caused 100% mortality)—it induced only transient weight loss (<14%) and achieved 100% survival. Furthermore, in a cutaneous infection model, VTT produced visible skin lesions at 2 × 10⁵ PFU, whereas NTV-ΔF1L-C7L caused no pathological damage even at 10⁷ PFU, equivalent to 500 times the pathogenic dose of VTT. This strikingly attenuated phenotype aligns with the expected consequence of F1L deletion: premature apoptosis of infected cells limits local viral replication and prevents spread to surrounding tissues.

Similar safety enhancements have been reported in other F1L-deleted poxvirus strains (33, 70), which is consistent with the notion that F1L’s anti-apoptotic activity may be central to its virulence function and that its deletion could represent a reliable and broadly applicable attenuation strategy. In contrast, prior VTT-based attenuation approaches, such as the C7L/K1L double-deletion strain (71) or the TK/F4L/B2R triple-deletion strain (69), were validated only at relatively low challenge doses (≤2.5 × 10⁶ PFU), falling short of probing the upper limits of viral pathogenicity. By increasing the challenge dose to 10⁷ PFU, our study recapitulates the attenuation conferred by F1L deletion and demonstrates a significantly wider safety margin under extreme conditions. Within the practical vaccination window (≤10⁶ PFU), NTV-ΔF1L-C7L matched the safety profile of parental NTV while delivering superior immunogenicity, providing a compelling preclinical foundation for its clinical translation.

This study has certain limitations. First, as with other live attenuated vaccines, the long-term immune protective efficacy of NTV-ΔF1L-C7L requires validation through challenge experiments conducted more than 4 months post-immunization in murine models (72). Second, before advancing to clinical trials, non-human primate studies are necessary to comprehensively evaluate the vaccine’s virulence and immunogenicity (63, 73, 74). These studies may provide valuable insights into the mechanisms underlying antigen-specific immune responses and the determinants of protective efficacy. Additionally, this investigation assessed only the nAb responses of NTV-ΔF1L-C7L against the MPXV Clade II strain. Although the neutralizing titers were comparable to those induced by VACV, the cross-neutralization potential against the more pathogenic Clade Ia and Ib MPXV strains remains to be determined. This assessment is critical for evaluating the breadth of the protective capacity (69). Despite these limitations, the attenuated live vaccine NTV-ΔF1L-C7L has several significant advantages. Its robust *in vitro* replication supports scalability for large-scale vaccine production. It has demonstrated strong immunoprotective efficacy in mice while maintaining a high safety margin. These findings establish a solid experimental foundation for the further development of NTV-ΔF1L-C7L as a next-generation VACV-based vector or candidate vaccine against mpox.

## MATERIALS AND METHODS

### Cells and viruses

All cell lines were obtained from the China Center for Type Culture Collection. Primary chicken embryo fibroblasts (CEFs) were prepared from 8-day-old chicken embryos. CEFs and Vero cells (African green monkey kidney) were cultured in Minimum Essential Medium (Gibco, USA) supplemented with 10% fetal bovine serum (FBS; Gibco) and 1% penicillin-streptomycin (Gibco). BHK-21 (Syrian hamster kidney), MRC-5 (human embryonic lung), HeLa (human cervical cancer), and BALB/c 3T3 (mouse embryonic fibroblasts) cell lines were cultured in Dulbecco’s Modified Eagle’s Medium (Gibco) containing 10% FBS. All cultures were incubated at 37°C in a humidified atmosphere with 5% CO_2_. MPXV (clade IIb lineage B.1.3) was propagated and titrated in Vero cells (Vero cells were chosen for viral titration due to their high permissiveness to orthopoxviruses) under Biosafety Level 3 (BSL-3) conditions. VTT, NTV, and VACV Western Reserve were preserved at −80°C in our laboratory.

### Western blot

HeLa cells were infected with the virus at an MOI of 5 or served as a control group with mock infection (cells harvested 24 h post-culture). Cells were harvested at 16 and 24 hpi. The cells were placed on ice and lysed with 0.5 mL of radioimmunoprecipitation assay buffer containing 1% phenylmethylsulfonyl fluoride for 10 min. The lysates were then separated via 10% sodium dodecyl sulfate–polyacrylamide gel electrophoresis and transferred to nitrocellulose membranes. The membranes were blocked overnight at 4°C with 5% skim milk in PBS with Tween 20 (PBST) and incubated overnight at 4°C with a series of primary antibodies. These antibodies included those targeting the apoptotic factors PARP (1:1,000, rabbit antiserum, Cell Signaling Technology), caspase 3 (1:300, rabbit antiserum, Cell Signaling Technology), caspase 8 (1:300, mouse antiserum, Cell Signaling Technology), caspase 9 (1:300, rabbit antiserum, Cell Signaling Technology), MPXV early protein E3 (1:500, mouse antiserum), and late proteins A29 (1:500), A35 (1:500), B6 (1:500), E8 (1:2,000), and M1 (1:500) (mouse antiserum). Subsequently, the membranes were incubated for 1 h at 25°C with horseradish peroxidase (HRP)-conjugated secondary antibodies (goat anti-mouse/rabbit, Zhongshan Goldenbridge). The protein signals were detected using the LI-COR Odyssey imaging system (USA).

### Immunostaining of virus plaques

Cell-to-cell transmission of the virus was assessed via immunostaining of viral plaques or foci, as previously described, with modifications (35). Briefly, target cells were seeded in 12-well plates and cultured to 90% –100% confluence, followed by infection with the virus at an MOI of 0.005. After 2 h of viral adsorption, the cells were washed three times with PBS and incubated at 37°C for 24, 48, or 72 h. The cells were fixed with cold methanol/acetone (1:1) for 20 min and incubated with rabbit anti-VTT serum (1:100 dilution in PBS containing 2% FBS) at 37°C for 1 h. After five washes with PBS, HRP-conjugated goat anti-rabbit and goat anti-mouse secondary antibodies (Zhongshan Goldenbridge Biotechnology, 1:5,000 dilution) were added and incubated for 1 h at 37°C. Following five additional washes, TrueBlue peroxidase substrate (KPL) was added for colorimetric detection of infected foci.

### Viral growth kinetics

Monolayers of Vero, MRC-5, CEF, HeLa, and BALB/c 3T3 cells were cultured in 12-well plates to evaluate the single-step and multi-step growth kinetics of the virus. For single-step growth kinetics (75), cells grown to over 90% confluence were infected with NTV-ΔF1L-C7L, NTV, or VTT at an MOI of 5. After a 2-h adsorption period (defined as 0 hpi), the cells were washed three times with PBS and cultured in fresh medium containing 2% FBS at 37°C with 5% CO₂. The cells and supernatants were harvested at 0, 2, 4, 8, and 12 hpi. For multi-step growth kinetics, the cells were infected at an MOI of 0.01, and samples were collected at 0, 24, 48, and 72 hpi. All harvested samples were subjected to three freeze–thaw cycles and briefly sonicated for 3–5 s. Virus titers were determined using the TCID₅₀ method with BHK-21 cells.

### Virulence assay in BALB/c mice

Virulence was assessed in female BALB/c mice (5–6 weeks old; Vital River Laboratory Animal Technology Co., Ltd., Beijing) using intranasal and tail scarification infection models. For intranasal challenge (*n* = 10) (57), mice anesthetized with isoflurane were inoculated bilaterally with 15 μL of viral suspension per nostril (30 μL total; containing 10^6^ or 10^7^ PFU). Body weight and survival were monitored daily for 14 days. Mice were euthanized upon >25% wt loss relative to baseline.

For cutaneous challenge (*n* = 3) (76), mice were anesthetized by intraperitoneal injection of ketamine (75 mg/kg) and xylazine (7.5 mg/kg) in PBS. A 10 μL droplet of viral suspension (2 × 10^5^ PFU) was placed ~1 cm from the base of the tail. A bifurcated needle (BD) was used to perform 25–30 skin punctures through the droplet in the direction of the tail tip. Mice were monitored daily for 14 days post-challenge for clinical symptoms, body weight, and survival.

### Immunization and challenge in mice

Female BALB/c mice (6–8 weeks old; *n* = 21) were randomly assigned to experimental groups. NTV or NTV-ΔF1L-C7L was administered via bilateral intramuscular injection (50 μL per gastrocnemius; total 100 μL containing 10^3^ –10^7^ PFU). A homologous booster was administered 2 weeks after primary immunization. The VTT group received a single dose (2 × 10^5^ PFU/10 μL) via tail scarification. Serum and splenocytes were collected on days 14 and 28 post-immunization to evaluate humoral and cellular immune responses, respectively. On day 42, all immunized mice were challenged intranasally with a lethal dose of VACV WR strain (10^7^ PFU, 25× LD_50_). For the reduced-dose challenge experiment, an additional cohort of mice was challenged with 10⁶ PFU VACV WR (2.5× LD₅₀) under the same immunization protocol. Naïve mice received PBS as a mock control. Mice were monitored daily for 14 days post-challenge for clinical symptoms, body weight, and survival. Euthanasia was performed when body weight loss exceeded 25%. On day 5 post-challenge, three mice per group were euthanized and necropsied. Lungs were harvested, fixed in 4% neutral-buffered formalin, embedded in paraffin, and sectioned for hematoxylin and eosin staining. Histopathological evaluation was performed by a veterinary pathologist using a standardized four-tier severity scoring system.

In the single-dose immunization and challenge protection study, female BALB/c mice (6–8 weeks old) were immunized with either NTV-ΔF1L-C7L (10^5^ PFU/100 μL) via a single intramuscular injection or VTT (2 × 10^5^ PFU/10 μL) via tail scarification. On day 42 post-immunization, mice were challenged intranasally with either 10⁷ PFU VACV WR (25× LD₅₀) or, in a separate experiment, with 10⁶ PFU VACV WR (2.5× LD₅₀). Mice were monitored daily for 7 days post-challenge for clinical symptoms, body weight, and survival. Euthanasia was performed when body weight loss exceeded 25%.

### ELISA

Serum IgG antibody titers were determined via ELISA. Purified VTT virions (10^5^ PFU/well) or proteins (E3, A29, A35, B6, E8, or M1) at 100 ng/well were diluted in 0.1 M carbonate coating buffer (pH 9.6) and adsorbed onto 96-well EIA/RIA plates (Corning Inc.) by overnight incubation at 4°C. The plates were washed three times with PBST and blocked at 37°C for 2 h. Heat-inactivated serum samples (56°C, 30 min) were serially diluted in PBST containing 2% FBS, and 100 μL aliquots were added per well for 1-h incubation at 37°C. Following five washes, HRP-conjugated anti-mouse IgG secondary antibody (1:10,000 in PBST with 2% FBS) was added for 1 h at 37°C. After additional washing, color development was performed using TMB substrate (Solarbio), and the reaction was terminated with 4.5 M sulfuric acid. Optical density was measured at 450 nm with a 630 nm reference using a MULTISKAN GO microplate reader (Thermo Fisher Scientific). Endpoint titers were defined as the highest serum dilution with an absorbance ratio (sample/blank) ≥ 2.1.

### Plaque reduction neutralization test

VTT EV was prepared as previously described (77). BHK cells were cultured in 225 cm² flasks. When the cell density exceeded 90%, the cells were infected at an MOI of 0.5. The culture medium containing EVs was collected at 48 hpi and centrifuged two times (450 × *g* , 8 min) to remove cells. The clarified supernatant was used immediately or stored at 4°C for up to 3 weeks. The titer of VTT EVs was determined on Vero cells, yielding approximately 1 × 10^5^ PFU/mL.

nAb titers were measured using the PRNT assay, as previously described (66). Briefly, heat-inactivated mouse serum was twofold serially diluted (1:20 –1:2,560) and incubated with 100 μL of VTT, VTT (EV), or MPXV (80 PFU) at 37°C for 2 h. The virus–serum mixtures were inoculated onto confluent Vero cell monolayers in 12-well plates and incubated at 37°C, 5% CO₂ for 48 hpi. Subsequently, the cells were fixed with 4 % formaldehyde (Solarbio, China) and stained with 0.1 % crystal violet. Neutralizing titers were defined as the highest serum dilution resulting in ≥50% plaque reduction (PRNT_50_) compared to virus-only controls.

### ELISpot assay

Mouse IFN-γ ELISpot assays were conducted using commercial kits (BD Biosciences) in accordance with the manufacturer’s instructions. Splenocytes were aseptically harvested and passed through 70 μm strainers to obtain single-cell suspensions. After erythrocyte lysis, the cells were resuspended at 2 × 10^5^ cells/well in 50 μL and stimulated with VACV-specific peptides E3 (140–148, VGPSNSPTF) or F2 (26–34, SPYAAGYDL) at 5 μg/mL (78). PMA (500 ng/mL) and ionomycin (10 μg/mL) served as positive controls. After 20 h of incubation at 37°C in 5 % CO₂, the cells were removed, the plates were washed with PBST, and detection antibody was added for 2 h at 37°C. Spots were developed using an AEC substrate and quantified using a Bioreader ELISpot analyzer (Biosys).

### Intracellular cytokine staining

ICS was performed on 2 × 10^6^ splenocytes stimulated with the E3 peptide (2 mg/mL) for 30 min at 37°C, followed by treatment with protein transport inhibitor (BD Pharmingen, 1:10 dilution) for an additional 10 h. Following centrifugation, live/dead discrimination was conducted using Fixable Viability Stain 780 (1:1,000 dilution, BD Pharmingen). Surface staining was performed using anti-CD3 (PerCP-Cy5.5), anti-CD4 (BV510), and anti-CD8 (FITC) antibodies. After fixation and permeabilization, intracellular staining was performed using anti-IFN-γ (BV421), IL-2 (PE), IL-4 (APC), and TNF-α (PE-Cy7) antibodies (all from BD Pharmingen). Samples were analyzed using a BD flow cytometer.

### Statistical analysis

Statistical analyses were performed using GraphPad Prism version 10.1.2 (GraphPad Software, Inc.). Data are expressed as mean ± standard error of the mean. Differences between groups were evaluated using one-way analysis of variance (nonparametric or mixed models). A *P*-value < 0.05 was considered significant.

## Data Availability

All data are available upon request to the corresponding author.

## SUPPLEMENTAL MATERIAL

Supplemental material jvi.00943-26-s0001.docx Supplemental methods, Fig. S1, and Tables S1 and S2.

ASM does not own the copyrights to Supplemental Material that may be linked to, or accessed through, an article. The authors have granted ASM a non-exclusive, world-wide license to publish the Supplemental Material files. Please contact the corresponding author directly for reuse.

## References

[1] Greseth MD , Traktman P . 2022 . The life cycle of the vaccinia virus genome . Annu Rev Virol 9 : 239 – 259 . doi:10.1146/annurev-virology-091919-104752 35584888

[2] World Health Organization . 2016 . Smallpox vaccines . Available from : https://www.who.int/news-room/feature-stories/detail/smallpox-vaccines

[3] Strassburg MA . 1982 . The global eradication of smallpox . Am J Infect Control 10 : 53 – 59 . doi:10.1016/0196-6553(82)90003-7 7044193

[4] Moss B . 1991 . Vaccinia virus: a tool for research and vaccine development . Science 252 : 1662 – 1667 . doi:10.1126/science.2047875 2047875

[5] Kaynarcalidan O , Moreno Mascaraque S , Drexler I . 2021 . Vaccinia virus: from crude smallpox vaccines to elaborate viral vector vaccine design . Biomedicines 9 : 1780 . doi:10.3390/biomedicines9121780 34944596 PMC8698642

[6] Zha M , Huang F , Li S , Wang Q , Tang Y . 2025 . Advances of oncolytic vaccinia viruses armed with interleukin in tumor therapy . Front Oncol 15 : 1594621 . doi:10.3389/fonc.2025.1594621 40469178 PMC12133479

[7] Fang Q , Yang L , Zhu W , Liu L , Wang H , Yu W , Xiao G , Tien P , Zhang L , Chen Z . 2005 . Host range, growth property, and virulence of the smallpox vaccine: vaccinia virus Tian Tan strain . Virology 335 : 242 – 251 . doi:10.1016/j.virol.2005.02.014 15840523

[8] Kretzschmar M , Wallinga J , Teunis P , Xing S , Mikolajczyk R . 2006 . Frequency of adverse events after vaccination with different vaccinia strains . PLoS Med 3 : e272 . doi:10.1371/journal.pmed.0030272 16933957 PMC1551910

[9] Gómez CE , Nájera JL , Jiménez EP , Jiménez V , Wagner R , Graf M , Frachette MJ , Liljeström P , Pantaleo G , Esteban M . 2007 . Head-to-head comparison on the immunogenicity of two HIV/AIDS vaccine candidates based on the attenuated poxvirus strains MVA and NYVAC co-expressing in a single locus the HIV-1BX08 gp120 and HIV-1(IIIB) Gag-Pol-Nef proteins of clade B . Vaccine 25 : 2863 – 2885 . doi:10.1016/j.vaccine.2006.09.090 17113200

[10] Sutter G , Staib C . 2003 . Vaccinia vectors as candidate vaccines: the development of modified vaccinia virus Ankara for antigen delivery . Curr Drug Targets Infect Disord 3 : 263 – 271 . doi:10.2174/1568005033481123 14529359

[11] Orsini D , Sartini M , Spagnolo AM , Cristina ML , Martini M . 2023 . Mpox: “the stigma is as dangerous as the virus”. Historical, social, ethical issues and future forthcoming . J Prev Med Hyg 64 : E398 – E404 . doi:10.15167/2421-4248/jpmh2023.64.4.3144 38379747 PMC10876020

[12] World Health Organization . 2025 . 2022-24 mpox (monkeypox) outbreak: global trends . Available from : https://worldhealthorg.shinyapps.io/mpx_global

[13] Fine PE , Jezek Z , Grab B , Dixon H . 1988 . The transmission potential of monkeypox virus in human populations . Int J Epidemiol 17 : 643 – 650 . doi:10.1093/ije/17.3.643 2850277

[14] Petersen BW , Harms TJ , Reynolds MG , Harrison LH . 2016 . Use of vaccinia virus smallpox vaccine in laboratory and health care personnel at risk for occupational exposure to orthopoxviruses - Recommendations of the advisory committee on immunization practices (ACIP), 2015 . MMWR Morb Mortal Wkly Rep 65 : 257 – 262 . doi:10.15585/mmwr.mm6510a2 26985679

[15] Rao AK , Petersen BW , Whitehill F , Razeq JH , Isaacs SN , Merchlinsky MJ , Campos-Outcalt D , Morgan RL , Damon I , Sánchez PJ , Bell BP . 2022 . Use of JYNNEOS (smallpox and monkeypox vaccine, live, nonreplicating) for preexposure vaccination of persons at risk for occupational exposure to orthopoxviruses: recommendations of the advisory committee on immunization practices - United States, 2022 . MMWR Morb Mortal Wkly Rep 71 : 734 – 742 . doi:10.15585/mmwr.mm7122e1 35653347 PMC9169520

[16] Anonymous . 2024 . WHO adds LC16m8 mpox vaccine to emergency use listing . Saudi Med J 45 : 1401 – 1402 . doi:10.15537/1658-3175.8502 39658109 PMC11629654

[17] Moss B . 1996 . Genetically engineered poxviruses for recombinant gene expression, vaccination, and safety . Proc Natl Acad Sci USA 93 : 11341 – 11348 . doi:10.1073/pnas.93.21.11341 8876137 PMC38059

[18] Zhu Y , Li D , Zhao R , Chen M , Li Y , Yang X , Mao H , Li X , Li Y , Shang C , Xia X . 2025 . Multiple gene-deletion vaccinia virus Tiantan strain against mpox . Virol J 22 : 17 . doi:10.1186/s12985-025-02629-6 39863910 PMC11762464

[19] Wen B , Deng Y , Chen H , Guan J , Chuai X , Ruan L , Kong W , Tan W . 2013 . The novel replication-defective vaccinia virus (Tiantan strain)-based hepatitis C virus vaccine induces robust immunity in macaques . Mol Ther 21 : 1787 – 1795 . doi:10.1038/mt.2013.122 23774793 PMC3776631

[20] Zhu L , Pan S , Huang B , Zhang J , Yang Z , Cong Z , Ma J , Qiu S , Liu Y , Zhang J , Li N , Lu J , Chen T , Hou Y , Zhang D , Wei Q , Li D , Tan W , Zhang Y , Xue J . 2025 . Tiantan vaccinia virus-based vaccine with promising safety provides sustained protection against mpox in non-human primates . Nat Commun 16 : 7183 . doi:10.1038/s41467-025-62594-0 40764511 PMC12325645

[21] Zhao Y , Zhao L , Huang P , Ren J , Zhang P , Tian H , Tan W . 2020 . Non-replicating vaccinia virus TianTan strain (NTV) translation arrest of viral late protein synthesis associated with anti-viral host factor SAMD9 . Front Cell Infect Microbiol 10 : 116 . doi:10.3389/fcimb.2020.00116 32266167 PMC7098914

[22] Staib C , Kisling S , Erfle V , Sutter G . 2005 . Inactivation of the viral interleukin 1beta receptor improves CD8+ T-cell memory responses elicited upon immunization with modified vaccinia virus Ankara . J Gen Virol 86 : 1997 – 2006 . doi:10.1099/vir.0.80646-0 15958679

[23] Clark RH , Kenyon JC , Bartlett NW , Tscharke DC , Smith GL . 2006 . Deletion of gene A41L enhances vaccinia virus immunogenicity and vaccine efficacy . J Gen Virol 87 : 29 – 38 . doi:10.1099/vir.0.81417-0 16361415

[24] García-Arriaza J , Nájera JL , Gómez CE , Tewabe N , Sorzano COS , Calandra T , Roger T , Esteban M . 2011 . A candidate HIV/AIDS vaccine (MVA-B) lacking vaccinia virus gene C6L enhances memory HIV-1-specific T-cell responses . PLoS One 6 : e24244 . doi:10.1371/journal.pone.0024244 21909386 PMC3164197

[25] Tartaglia J , Perkus ME , Taylor J , Norton EK , Audonnet JC , Cox WI , Davis SW , van der Hoeven J , Meignier B , Riviere M . 1992 . NYVAC: a highly attenuated strain of vaccinia virus . Virology 188 : 217 – 232 . doi:10.1016/0042-6822(92)90752-b 1566575

[26] Antoine G , Scheiflinger F , Dorner F , Falkner FG . 1998 . The complete genomic sequence of the modified vaccinia Ankara strain: comparison with other orthopoxviruses . Virology 244 : 365 – 396 . doi:10.1006/viro.1998.9123 9601507

[27] Nájera JL , Gómez CE , García-Arriaza J , Sorzano CO , Esteban M . 2010 . Insertion of vaccinia virus C7L host range gene into NYVAC-B genome potentiates immune responses against HIV-1 antigens . PLoS One 5 : e11406 . doi:10.1371/journal.pone.0011406 20613977 PMC2894869

[28] Nájera JL , Gómez CE , Domingo-Gil E , Gherardi MM , Esteban M . 2006 . Cellular and biochemical differences between two attenuated poxvirus vaccine candidates (MVA and NYVAC) and role of the C7L gene . J Virol 80 : 6033 – 6047 . doi:10.1128/JVI.02108-05 16731942 PMC1472566

[29] Volz A , Jany S , Freudenstein A , Lantermann M , Ludwig H , Sutter G . 2018 . E3L and F1L gene functions modulate the protective capacity of modified vaccinia virus Ankara immunization in murine model of human smallpox . Viruses 10 : 21 . doi:10.3390/v10010021 29300297 PMC5795434

[30] Hornemann S , Harlin O , Staib C , Kisling S , Erfle V , Kaspers B , Häcker G , Sutter G . 2003 . Replication of modified vaccinia virus Ankara in primary chicken embryo fibroblasts requires expression of the interferon resistance gene E3L . J Virol 77 : 8394 – 8407 . doi:10.1128/jvi.77.15.8394-8407.2003 12857909 PMC165266

[31] Ludwig H , Mages J , Staib C , Lehmann MH , Lang R , Sutter G . 2005 . Role of viral factor E3L in modified vaccinia virus ankara infection of human HeLa cells: regulation of the virus life cycle and identification of differentially expressed host genes . J Virol 79 : 2584 – 2596 . doi:10.1128/JVI.79.4.2584-2596.2005 15681458 PMC546556

[32] Stewart TL , Wasilenko ST , Barry M . 2005 . Vaccinia virus F1L protein is a tail-anchored protein that functions at the mitochondria to inhibit apoptosis . J Virol 79 : 1084 – 1098 . doi:10.1128/JVI.79.2.1084-1098.2005 15613337 PMC538563

[33] Gerlic M , Faustin B , Postigo A , Yu EC-W , Proell M , Gombosuren N , Krajewska M , Flynn R , Croft M , Way M , Satterthwait A , Liddington RC , Salek-Ardakani S , Matsuzawa S , Reed JC . 2013 . Vaccinia virus F1L protein promotes virulence by inhibiting inflammasome activation . Proc Natl Acad Sci USA 110 : 7808 – 7813 . doi:10.1073/pnas.1215995110 23603272 PMC3651467

[34] Perdiguero B , Gómez CE , Nájera JL , Sorzano COS , Delaloye J , González-Sanz R , Jiménez V , Roger T , Calandra T , Pantaleo G , Esteban M . 2012 . Deletion of the viral anti-apoptotic gene F1L in the HIV/AIDS vaccine candidate MVA-C enhances immune responses against HIV-1 antigens . PLoS One 7 : e48524 . doi:10.1371/journal.pone.0048524 23119046 PMC3485360

[35] Carroll MW , Moss B . 1997 . Host range and cytopathogenicity of the highly attenuated MVA strain of vaccinia virus: propagation and generation of recombinant viruses in a nonhuman mammalian cell line . Virology 238 : 198 – 211 . doi:10.1006/viro.1997.8845 9400593

[36] Morino E , Mine S , Tomita N , Uemura Y , Shimizu Y , Saito S , Suzuki T , Okumura N , Iwasaki H , Terada J , et al. . 2024 . Mpox neutralizing antibody response to LC16m8 vaccine in healthy adults . NEJM Evid 3 : EVIDoa2300290 . doi:10.1056/EVIDoa2300290 38411447

[37] Ren SY , Li J , Gao RD . 2022 . 2022 Monkeypox outbreak: why is it a public health emergency of international concern? What can we do to control it? World J Clin Cases 10 : 10873 – 10881 . doi:10.12998/wjcc.v10.i30.10873 36338228 PMC9631123

[38] Kenner J , Cameron F , Empig C , Jobes DV , Gurwith M . 2006 . LC16m8: an attenuated smallpox vaccine . Vaccine 24 : 7009 – 7022 . doi:10.1016/j.vaccine.2006.03.087 17052815 PMC7115618

[39] Jordan I , Sandig V . 2014 . Matrix and backstage: cellular substrates for viral vaccines . Viruses 6 : 1672 – 1700 . doi:10.3390/v6041672 24732259 PMC4014716

[40] Terajima M , Urban SL , Leporati AM . 2013 . The N-terminus of vaccinia virus host range protein C7L is essential for function . Virus Genes 46 : 20 – 27 . doi:10.1007/s11262-012-0822-x 23001690 PMC3546155

[41] Meng X , Chao J , Xiang Y . 2008 . Identification from diverse mammalian poxviruses of host-range regulatory genes functioning equivalently to vaccinia virus C7L . Virology 372 : 372 – 383 . doi:10.1016/j.virol.2007.10.023 18054061 PMC2276162

[42] Riad S , Xiang Y , AlDaif B , Mercer AA , Fleming SB . 2020 . Rescue of a vaccinia virus mutant lacking IFN resistance genes K1L and C7L by the parapoxvirus Orf virus . Front Microbiol 11 : 1797 . doi:10.3389/fmicb.2020.01797 32903701 PMC7438785

[43] Meng X , Schoggins J , Rose L , Cao J , Ploss A , Rice CM , Xiang Y . 2012 . C7L family of poxvirus host range genes inhibits antiviral activities induced by type I interferons and interferon regulatory factor 1 . J Virol 86 : 4538 – 4547 . doi:10.1128/JVI.06140-11 22345458 PMC3318637

[44] Meng X , Jiang C , Arsenio J , Dick K , Cao J , Xiang Y . 2009 . Vaccinia virus K1L and C7L inhibit antiviral activities induced by type I interferons . J Virol 83 : 10627 – 10636 . doi:10.1128/JVI.01260-09 19656868 PMC2753149

[45] Sivan G , Ormanoglu P , Buehler EC , Martin SE , Moss B . 2015 . Identification of restriction factors by human genome-wide RNA interference screening of viral host range mutants exemplified by discovery of SAMD9 and WDR6 as inhibitors of the vaccinia virus K1L-C7L-mutant . mBio 6 : e01122 . doi:10.1128/mBio.01122-15 26242627 PMC4526713

[46] Meng X , Zhang F , Yan B , Si C , Honda H , Nagamachi A , Sun LZ , Xiang Y . 2018 . A paralogous pair of mammalian host restriction factors form a critical host barrier against poxvirus infection . PLoS Pathog 14 : e1006884 . doi:10.1371/journal.ppat.1006884 29447249 PMC5831749

[47] Backes S , Sperling KM , Zwilling J , Gasteiger G , Ludwig H , Kremmer E , Schwantes A , Staib C , Sutter G . 2010 . Viral host-range factor C7 or K1 is essential for modified vaccinia virus Ankara late gene expression in human and murine cells, irrespective of their capacity to inhibit protein kinase R-mediated phosphorylation of eukaryotic translation initiation factor 2alpha . J Gen Virol 91 : 470 – 482 . doi:10.1099/vir.0.015347-0 19846675

[48] Rao AK , Minhaj FS , Carter RJ , Duffy J , Satheshkumar PS , Delaney KP , Quilter LAS , Kachur RE , McLean C , Moulia DL , Kuhar DT , de Perio MA , Spicknall IH , Bell BP , Sánchez PJ , Hutson CL , Cohn AC . 2025 . Use of JYNNEOS (smallpox and mpox vaccine, live, nonreplicating) for persons aged ≥18 years at risk for mpox during an mpox outbreak: recommendations of the advisory committee on immunization practices - United States, 2023 . MMWR Morb Mortal Wkly Rep 74 : 385 – 392 . doi:10.15585/mmwr.mm7422a3 40531798 PMC12176103

[49] Dimitrov D , Adamson B , Matrajt L . 2023 . Evaluation of mpox vaccine dose-sparing strategies . PNAS Nexus 2 :p gad095 . doi:10.1093/pnasnexus/pgad095 PMC10154907 37152676

[50] Gahr S , Perinetti Casoni G , Falk-Paulsen M , Maschkowitz G , Bryceson YT , Voss M . 2023 . Viral host range factors antagonize pathogenic SAMD9 and SAMD9L variants . Exp Cell Res 425 : 113541 . doi:10.1016/j.yexcr.2023.113541 36894052

[51] Edghill-Smith Y , Golding H , Manischewitz J , King LR , Scott D , Bray M , Nalca A , Hooper JW , Whitehouse CA , Schmitz JE , Reimann KA , Franchini G . 2005 . Smallpox vaccine-induced antibodies are necessary and sufficient for protection against monkeypox virus . Nat Med 11 : 740 – 747 . doi:10.1038/nm1261 15951823

[52] Panchanathan V , Chaudhri G , Karupiah G . 2010 . Antiviral protection following immunization correlates with humoral but not cell-mediated immunity . Immunol Cell Biol 88 : 461 – 467 . doi:10.1038/icb.2009.110 20066003

[53] Belyakov IM , Earl P , Dzutsev A , Kuznetsov VA , Lemon M , Wyatt LS , Snyder JT , Ahlers JD , Franchini G , Moss B , Berzofsky JA . 2003 . Shared modes of protection against poxvirus infection by attenuated and conventional smallpox vaccine viruses . Proc Natl Acad Sci USA 100 : 9458 – 9463 . doi:10.1073/pnas.1233578100 12869693 PMC170940

[54] Xu R , Johnson AJ , Liggitt D , Bevan MJ . 2004 . Cellular and humoral immunity against vaccinia virus infection of mice . J Immunol 172 : 6265 – 6271 . doi:10.4049/jimmunol.172.10.6265 15128815

[55] Moss B . 2011 . Smallpox vaccines: targets of protective immunity . Immunol Rev 239 : 8 – 26 . doi:10.1111/j.1600-065X.2010.00975.x 21198662 PMC3074351

[56] Cheng X , Wang Y , Huang B , Bing J , Wang T , Han R , Huo S , Sun S , Zhao L , Shu C , Deng Y , Tan W . 2025 . Rational mpox vaccine design: immunogenicity and protective effect of individual and multicomponent proteins in mice . Emerg Microbes Infect 14 : 2482702 . doi:10.1080/22221751.2025.2482702 40105863 PMC11951338

[57] Melamed S , Wyatt LS , Kastenmayer RJ , Moss B . 2013 . Attenuation and immunogenicity of host-range extended modified vaccinia virus Ankara recombinants . Vaccine 31 : 4569 – 4577 . doi:10.1016/j.vaccine.2013.07.057 23928462 PMC3787882

[58] Meseda CA , Garcia AD , Kumar A , Mayer AE , Manischewitz J , King LR , Golding H , Merchlinsky M , Weir JP . 2005 . Enhanced immunogenicity and protective effect conferred by vaccination with combinations of modified vaccinia virus Ankara and licensed smallpox vaccine Dryvax in a mouse model . Virology 339 : 164 – 175 . doi:10.1016/j.virol.2005.06.002 15993917

[59] Su W , Zhao T , Ren X , Li S , Huang Q , Liu J , Zhang X , Ge Z , Wei J . 2026 . Protective efficacy of a genetically modified attenuated vaccinia virus Tiantan strain against monkeypox virus challenge in a small animal model . J Virol 100 : e0184325 . doi:10.1128/jvi.01843-25 41589837 PMC12911895

[60] Li E , Yang Q , Xie W , Gong Q , Guo X , Zhou J , Zhang J , Chuai X , Wang Y , Chiu S . 2025 . An mpox quadrivalent mRNA vaccine elicits sustained and protective immunity in mice against lethal vaccinia virus challenge . Emerg Microbes Infect 14 : 2447619 . doi:10.1080/22221751.2024.2447619 39745170 PMC11758793

[61] Zuo J , Wu J , Zhang Z , Long J , Yu C , Liao Y , Zhang H , Yang J . 2025 . An mpox multi-antigen-tandem bivalent mRNA candidate vaccine effectively protects mice against the vaccinia virus . Vaccines (Basel) 13 : 374 . doi:10.3390/vaccines13040374 40333228 PMC12031407

[62] Cotter CA , Ignacio MA , Americo JL , Earl PL , Mucker EM , Frey TR , Carfi A , Hooper JW , Freyn AW , Moss B . 2024 . Mpox mRNA-1769 vaccine inhibits orthopoxvirus replication at intranasal, intrarectal, and cutaneous sites of inoculation . NPJ Vaccines 9 : 256 . doi:10.1038/s41541-024-01052-2 39719481 PMC11668852

[63] Mucker EM , Freyn AW , Bixler SL , Cizmeci D , Atyeo C , Earl PL , Natarajan H , Santos G , Frey TR , Levin RH , et al. . 2024 . Comparison of protection against mpox following mRNA or modified vaccinia Ankara vaccination in nonhuman primates . Cell 187 : 5540 – 5553 . doi:10.1016/j.cell.2024.08.043 39236707

[64] Kobiyama K , Utsumi D , Kaku Y , Sasaki E , Yasui F , Okamura T , Onodera T , Tobuse AJ , Sakkour A , Amiry AF , Hayashi T , Temizoz B , Liu K , Negishi H , Toyama-Sorimachi N , Kohara M , Sawasaki T , Takagi J , Sato K , Takahashi Y , Yasutomi Y , Ishii KJ . 2025 . Immunological analysis of LC16m8 vaccine: preclinical and early clinical insights into mpox . EBioMedicine 115 : 105703 . doi:10.1016/j.ebiom.2025.105703 40239465 PMC12020844

[65] Liu Y , Lv W , Shan P , Li D , Wu YQ , Wang YC , Li YY , Liu Q , Wang JS , Hao YL , Liu Y , Huang WJ , Ren L , Wang SH , Li TS , Xu J , Shao YM . 2025 . Safety and immunogenicity of an HIV vaccine trial with DNA prime and replicating vaccinia boost . Sig Transduct Target Ther 10 : 208 . doi:10.1038/s41392-025-02259-y PMC12217030 40593459

[66] Li E , Guo X , Hong D , Gong Q , Xie W , Li T , Wang J , Chuai X , Chiu S . 2023 . Duration of humoral immunity from smallpox vaccination and its cross-reaction with Mpox virus . Sig Transduct Target Ther 8 : 350 . doi:10.1038/s41392-023-01574-6 PMC10502045 37709783

[67] Payne AB , Ray LC , Kugeler KJ , Fothergill A , White EB , Canning M , Farrar JL , Feldstein LR , Gundlapalli AV , Houck K , Kriss JL , Lewis NM , Sims E , Smith DK , Spicknall IH , Nakazawa Y , Damon IK , Cohn AC , Payne DC . 2022 . Incidence of monkeypox among unvaccinated persons compared with persons receiving ≥1 JYNNEOS vaccine dose - 32 U.S. jurisdictions, July 31-September 3, 2022 . MMWR Morb Mortal Wkly Rep 71 : 1278 – 1282 . doi:10.15585/mmwr.mm7140e3 36201401 PMC9541026

[68] Wolff Sagy Y , Zucker R , Hammerman A , Markovits H , Arieh NG , Abu Ahmad W , Battat E , Ramot N , Carmeli G , Mark-Amir A , Wagner-Kolasko G , Duskin-Bitan H , Yaron S , Peretz A , Arbel R , Lavie G , Netzer D . 2023 . Real-world effectiveness of a single dose of mpox vaccine in males . Nat Med 29 : 748 – 752 . doi:10.1038/s41591-023-02229-3 36720271 PMC9930701

[69] Xu F , Huang Y , Hou Y , Xie Y , Huang B , Zhao F , Gao Z , Chen C , Wang J , Mei S , Hu Y , Wang L , Wei L , Zhang J , Li N , Cong Z , Ma J , Zhu L , Chen T , Lu J , Wei Q , Tan W , Xue J , Guo F . 2025 . A new attenuated and highly immunogenic orthopoxvirus vaccine protects against mpox in mice and macaques . NPJ Vaccines 10 : 134 . doi:10.1038/s41541-025-01193-y 40593776 PMC12214906

[70] Pelin A , Foloppe J , Petryk J , Singaravelu R , Hussein M , Gossart F , Jennings VA , Stubbert LJ , Foster M , Storbeck C , Postigo A , Scut E , Laight B , Way M , Erbs P , Le Boeuf F , Bell JC . 2019 . Deletion of apoptosis inhibitor F1L in vaccinia virus increases safety and oncolysis for cancer therapy . Mol Ther Oncolytics 14 : 246 – 252 . doi:10.1016/j.omto.2019.06.004 31428674 PMC6695278

[71] Liu Z , Wang S , Zhang Q , Tian M , Hou J , Wang R , Liu C , Ji X , Liu Y , Shao Y . 2013 . Deletion of C7L and K1L genes leads to significantly decreased virulence of recombinant vaccinia virus TianTan . PLoS One 8 : e68115 . doi:10.1371/journal.pone.0068115 23840887 PMC3698190

[72] Cotter CA , Ignacio MA , Americo JL , Earl PL , Mucker EM , Hooper JW , Frey TR , Carfi A , Freyn AW , Moss B . 2024 . Mpox mRNA-1769 vaccine inhibits orthopoxvirus replication at intranasal, intrarectal, and cutaneous sites of inoculation . bioRxiv : 2024.09.19.613928 . doi:10.1101/2024.09.19.613928 PMC11668852 39719481

[73] Zuiani A , Dulberger CL , De Silva NS , Marquette M , Lu Y-J , Palowitch GM , Dokic A , Sanchez-Velazquez R , Schlatterer K , Sarkar S , et al. . 2024 . A multivalent mRNA monkeypox virus vaccine (BNT166) protects mice and macaques from orthopoxvirus disease . Cell 187 : 1363 – 1373 . doi:10.1016/j.cell.2024.01.017 38366591

[74] Ye Q , Zhang D , Zhang RR , Xu Q , Huang XY , Huang B , Sun MX , Cong Z , Zhu L , Ma J , et al. . 2024 . A penta-component mpox mRNA vaccine induces protective immunity in nonhuman primates . Nat Commun 15 : 10611 . doi:10.1038/s41467-024-54909-4 39639037 PMC11621575

[75] Harrison K , Haga IR , Pechenick Jowers T , Jasim S , Cintrat JC , Gillet D , Schmitt-John T , Digard P , Beard PM . 2016 . Vaccinia virus uses retromer-independent cellular retrograde transport pathways to facilitate the wrapping of intracellular mature virions during virus morphogenesis . J Virol 90 : 10120 – 10132 . doi:10.1128/JVI.01464-16 27581988 PMC5105650

[76] Melamed S , Paran N , Katz L , Ben-Nathan D , Israely T , Schneider P , Levin R , Lustig S . 2007 . Tail scarification with vaccinia virus Lister as a model for evaluation of smallpox vaccine potency in mice . Vaccine 25 : 7743 – 7753 . doi:10.1016/j.vaccine.2007.09.023 17928110

[77] Benhnia MR-E-I , McCausland MM , Moyron J , Laudenslager J , Granger S , Rickert S , Koriazova L , Kubo R , Kato S , Crotty S . 2009 . Vaccinia virus extracellular enveloped virion neutralization *in vitro* and protection *in vivo* depend on complement . J Virol 83 : 1201 – 1215 . doi:10.1128/JVI.01797-08 19019965 PMC2620895

[78] Tscharke DC , Woo W-P , Sakala IG , Sidney J , Sette A , Moss DJ , Bennink JR , Karupiah G , Yewdell JW . 2006 . Poxvirus CD8^+^ T-cell determinants and cross-reactivity in BALB/c mice . J Virol 80 : 6318 – 6323 . doi:10.1128/JVI.00427-06 16775319 PMC1488955

