# Supplementary material for: Enhanced replication and immunogenicity of a genome-modified non-replicating vaccinia virus Tiantan strain for robust low-dose protection in mice
Source: J Virol. 2026 Jul 31;100(8):e00943-26. doi: 10.1128/jvi.00943-26 (PMC13483249; doi:10.1128/jvi.00943-26)
Supplement: Supplementary file 1 [file jvi.00943-26-s0001.docx]

**Supplemental Materials**

**Plasmid Construction**

A Cas9-GFP–expressing plasmid (pX458del-gRNA), previously constructed and stored in our laboratory, was used for CRISPR/Cas9-mediated gene editing. A guide RNA (gRNA) targeting the *F1L* gene of the NTV genome was designed using the CRISPOR tool (http://crispor.tefor.net/). The target sequence (5'-CACCGTGTTAGGGATGGCATTATC-3') was cloned into the *Bbs*l site of the pX458del-gRNA plasmid, resulting in the plasmid pX458del-616gRNA. The construct was sequence-verified by GenScript Biotech (Nanjing, China). To generate the donor plasmid for homologous recombination, a transfer vector (pUC57-ΔF1L-LacZ-C7L) was constructed as follows:

1. Insertion of homologous arms: A 666 bp upstream and 719 bp downstream region flanking the *F1L* gene was PCR-amplified from NTV genomic DNA and cloned into the PUC57 vector.
2. Promoter insertion: The vaccinia virus P7.5 promoter (an early/late promoter) was inserted between the homologous arms to facilitate gene expression, yielding an intermediate plasmid, pUC57-ΔF1L.
3. LacZ insertion: The LacZ reporter gene (driven by the H6R promoter) was excised from pJSEDH6LacZ using *Xma*I and cloned into pUC57-ΔF1L to generate pUC57-ΔF1L-LacZ.
4. *C7L* gene insertion: The *C7L* gene was PCR-amplified from VTT genomic DNA, digested with *Bam*HI-HF (NEB, USA), and cloned into the above vector to yield the final recombinant transfer vector pUC57-ΔF1L-LacZ-C7L.

All plasmids were confirmed by via Sanger sequencing before use.

**Recombinant virus construction**

The recombinant virus NTV-∆F1L-C7L was engineered using CRISPR/Cas9-mediated homologous recombination for its high efficiency and precision in vaccinia genome editing(1). Briefly, pX458del-616gRNA plasmid was transfected into BHK-21 cells using X-tremeGENE HP DNA transfection reagent (Roche, USA), followed by overnight incubation at 37 °C with 5% CO₂. Subsequently, the cells were infected with NTV at a multiplicity of infection (MOI) of 0.01. After 2 h of incubation at 37°C, the cells were washed three times with phosphate-buffered saline (PBS). Thereafter, the donor plasmid pUC57-∆F1L-LacZ-C7L was transfected into the infected cells. After 48 h, supernatants were harvested following three freeze–thaw cycles. Viral progeny were inoculated onto BHK-21 cell monolayers in six-well plates. After 48 h of incubation at 37°C, plaques were overlaid with 1.3% agarose-containing Dulbecco's Modified Eagle’s Medium supplemented with 50 μg/mL neutral red, 200 μg/mL X-gal (a substrate for β-galactosidase encoded by lacZ, producing an insoluble blue precipitate), and 2% FBS. Blue plaques indicative of LacZ expression were selected and subjected to 3–5 rounds of plaque purification to isolate NTV-∆F1L-C7L.

**PCR Identification of Genetic Recombination of NTV-ΔF1L-C7L**

Genomic modifications in the recombinant virus were determined using PCR with primers flanking the target regions (Fig 1A). The PCR primers used for the *F1L* deletion were primer1-F: 5′-TTATCCTATCATGTATTTGAGAGT-3′ and Primer1-R: 5′-ATGTTGTCGATGTTTATGTGTAAT-3′ (antisense); for the insertion of the *C7L* gene, Primer2-F: 5′-ACCATGGGTATACAGCACGAATTCG-3′ and Primer2-R: 5′-TCCATGGACTCATAATCTCTATACG-3′. PCR products were analyzed via agarose gel electrophoresis.

**Expression of Proteins Related to NTV-ΔF1L-C7L**

BHK-21 cells were infected at an MOI of 5 or mock-infected as controls. At 16 h post-infection, the cells were lysed on ice with 0.5 mL of radioimmunoprecipitation assay buffer containing 1% phenylmethylsulfonyl fluoride for 10 min. Lysates were separated using 10% sodium dodecyl sulfate–polyacrylamide gel electrophoresis and transferred onto nitrocellulose membranes. After blocking with 5% skim milk in PBST overnight at 4 °C, the membranes were incubated overnight at 4 °C with primary antibodies against F1 (mouse antiserum, 1:200), C7 (mouse antiserum, 1:50), and E3 (mouse antiserum, 1:200). Subsequently, the membranes were incubated with horseradish peroxidase (HRP)-conjugated secondary antibodies for 1 h at 25 °C. Protein signals were detected using the LI-COR Odyssey imaging system (USA).

**Clinical scoring for morbidity assessment after VACV challenge**

Clinical scoring after viral challenge was adapted from the murine sepsis score system described previously (2). Mice were evaluated at two time windows: days 1–3 and 4–7 post‑challenge. At each time point, three blinded observers independently scored the animals. Six parameters were scored: appearance, level of consciousness, activity, response to stimulus, eyes, and respiration rate. Each parameter was graded on a scale of 0–4, with the following specific criteria:

Appearance: 0 = smooth coat; 1 = patches of piloerection; 2 = majority of back piloerected; 3 = puffy appearance (with or without piloerection); and 4 = emaciated.

Level of consciousness: 0 = active; 1 = active but avoids standing upright; 2 = noticeably slowed activity, still ambulant; 3 = impaired activity, moves only when provoked, possible tremor; and 4 = severely impaired, remains stationary when provoked, cannot right itself.

Activity: 0 = normal (eating, drinking, climbing, running); 1 = slightly suppressed (moving around bottom of cage); 2 = suppressed (stationary with occasional investigative movements); 3 = no activity (stationary); and 4 = no activity with tremors (especially hind legs).

Response to stimulus: 0 = immediate response to auditory stimulus or touch; 1 = slow or no response to sound, strong response to touch (moves to escape); 2 = no response to sound, moderate response to touch (moves a few steps); 3 = no response to sound, mild response to touch (no locomotion); and 4 = no response to sound, little or no response to touch, and cannot right itself.

Eyes: 0 = open; 1 = not fully open, possibly with secretions; 2 = at least half closed, possibly with secretions; 3 = half closed or more, possibly with secretions; and 4 = closed or milky.

Respiration rate: 0 = normal rapid mouse respiration; 1 = slightly decreased; 2 = moderately reduced (rate quantifiable by eye at upper range); 3 = severely reduced (rate easily countable, ~0.5 s between breaths); and 4 = extremely reduced (>1 s between breaths).

The total clinical score was calculated as the sum of the six individual scores (maximum 24). Higher scores indicate more severe illness. Severity grading (total score): 0–4 (normal), 5–9 (mild), 10–15 (moderate), 16–20 (severe), and >20 (moribund). Mice reaching a humane endpoint (score ≥ 16 or body weight loss > 25% of initial weight) were euthanized.

**Supplemental Results**

**Construction and identification of NTV-ΔF1L-C7L**

CRISPR/Cas9-mediated gene editing combined with homologous recombination was employed to construct the recombinant vaccinia virus NTV-ΔF1L-C7L (FIG S1 A). PCR and western blot analyses confirmed the recombinant virus’s integrity, using NTV and VTT as controls. PCR showed no amplification of the *F1L* gene (738 bp) in NTV-ΔF1L-C7L (FIG S1 B), while a specific C7L band (452 bp) was detected in both NTV-ΔF1L-C7L and VTT (FIG S1 C), confirming successful deletion of *F1L* and insertion of *C7L*. Western blot analysis validated protein expression (FIG S1 1D). All viruses expressed the 22 kDa E3 early protein, an infection marker(1). Notably, NTV-ΔF1L-C7L lacked the 27 kDa F1 early protein, consistent with the deletion of *F1L*, and uniquely expressed the 17 kDa C7 late protein, which was undetectable in NTV, confirming functional *C7L* expression.

**Supplemental Figures**

FIG S1 Construction and identification of NTV-ΔF1L-C7L. (A) Schematic representation of the recombinant structure of NTV-ΔF1L-C7L. NTV was used as the target virus for modification. Under the guidance of gRNA in the CRISPR/Cas9 system, the recombinant plasmid pUC57-ΔF1L-LacZ-C7L was subjected to targeted homologous recombination with NTV. The recombination occurred in the F region of the NTV genome, where the *F1L* gene was deleted and the *C7L* gene was inserted at the same position to construct NTV-ΔF1L-C7L. (B and C) PCR detection of the deletion of the *F1L* gene (738 bp) and insertion of the *C7L* gene (452 bp) in the NTV-ΔF1L-C7L genome. Primers Primer1-F/R were designed at both ends of the *F1L* gene for the identification of the deletion of the *F1L* gene (738 bp) and Primers Primer2-F/R were designed at both ends of the *C7L* gene for the identification of the insertion of the *C7L* gene (452 bp) in the recombinant virus (marked in Fig 1A). (D) The expression of F1 and C7 proteins in NTV-ΔF1L-C7L was identified by Western blot. E3 and F1 are early proteins with sizes of 22 and 27 kDa, respectively, while C7 is a late protein with a size of 17 kDa. β-actin, which has a size of 42 kDa, was used as the internal control protein.

**Supplemental Tables**

**TABLE S1 Clinical scores after 10⁶ PFU VACV WR challenge following two-dose immunization**

| Group | Immunization dose (PFU) | Time (days) | Appearance | Consciousness | Activity | Response to stimulus | Eyes | Respiration rate | Total  Score ^a^ |
| --- | --- | --- | --- | --- | --- | --- | --- | --- | --- |
| Naïve | - | 1–3 | 0 | 0 | 0 | 0.2 | 0.2 | 0 | 0.4 |
| Naïve | - | 4–7 | 0 | 0 | 0.2 | 0.2 | 0 | 0 | 0.4 |
| NTV | 10³ | 1–3 | 2.4 | 2.4 | 2.8 | 2.2 | 3 | 2.4 | 15.2 |
| NTV |  | 4–7 | 2.8 | 3 | 2.8 | 2.4 | 3.4 | 2.8 | 17.2 |
| NTV | 10⁴ | 1–3 | 2.2 | 2.2 | 2.2 | 2 | 2.2 | 2.4 | 13.2 |
| NTV |  | 4–7 | 1.6 | 1.6 | 1.4 | 1.8 | 1.8 | 1.8 | 10.0 |
| NTV | 10⁵ | 1–3 | 1.6 | 1.6 | 1.4 | 1.6 | 1.4 | 1.4 | 9.0 |
| NTV |  | 4–7 | 1.0 | 1.2 | 0.8 | 1.0 | 1.0 | 0.8 | 5.8 |
| NTV-ΔF1L-C7L | 10³ | 1–3 | 1.6 | 1.4 | 1.2 | 1.0 | 1.0 | 0.8 | 7.0 |
| NTV-ΔF1L-C7L |  | 4–7 | 1.0 | 0.8 | 0.8 | 0.8 | 0.6 | 0.6 | 4.6 |
| NTV-ΔF1L-C7L | 10⁴ | 1–3 | 1.4 | 1.4 | 1.0 | 0.6 | 0.8 | 0.4 | 5.6 |
| NTV-ΔF1L-C7L |  | 4–7 | 1.0 | 1.0 | 0.8 | 0.6 | 0.2 | 0.2 | 3.8 |
| NTV-ΔF1L-C7L | 10⁵ | 1–3 | 1.0 | 0.8 | 0.8 | 0.2 | 0.2 | 0.6 | 3.6 |
| NTV-ΔF1L-C7L |  | 4–7 | 0.6 | 0.6 | 0.4 | 0 | 0.2 | 0.2 | 2.0 |
| VTT | 2×10⁵ | 1–3 | 1.4 | 1.4 | 1.0 | 0.8 | 0.8 | 0.4 | 5.8 |
| VTT |  | 4–7 | 0.8 | 0.8 | 0.6 | 0.6 | 0.6 | 0.2 | 3.6 |
| PBS | - | 1–3 | 2.4 | 2.4 | 2.0 | 2.0 | 2.0 | 2.2 | 13.0 |
| PBS | - | 4–7 | 3.6 | 3.6 | 3.2 | 3.4 | 3.2 | 3.6 | 20.6 |

^a^ *Severity grading (total score): 0-4 (Normal), 5-9 (Mild), 10-15 (Moderate), 16-20 (Severe), >20 (Moribund).*

**TABLE S2 Clinical scores after 10⁶ PFU VACV WR challenge following single-dose immunization**

| Group | Immunization dose (PFU) | Time (days) | Appearance | Consciousness | Activity | Response to stimulus | Eyes | Respiration rate | Total  Score |
| --- | --- | --- | --- | --- | --- | --- | --- | --- | --- |
| Naïve | - | 1–3 | 0.2 | 0 | 0.2 | 0 | 0 | 0.2 | 0.6 |
| Naïve | - | 4–7 | 0 | 0 | 0.2 | 0 | 0 | 0 | 0.2 |
| NTV-ΔF1L-C7L | 10⁵ | 1–3 | 1.4 | 1.6 | 0.8 | 0.8 | 0.8 | 0.4 | 5.8 |
| NTV-ΔF1L-C7L |  | 4–7 | 1.0 | 1.0 | 1.0 | 0.6 | 0.2 | 0.2 | 4.0 |
| VTT | 2×10⁵ | 1–3 | 1.6 | 1.4 | 1.2 | 0.8 | 0.6 | 0.4 | 6.0 |
| VTT |  | 4–7 | 0.8 | 0.6 | 0.8 | 0.6 | 0.4 | 0.2 | 3.4 |
| PBS | - | 1–3 | 2.2 | 2.4 | 2.0 | 2.2 | 2.0 | 2.0 | 12.8 |
| PBS | - | 4–7 | 3.4 | 3.6 | 3.6 | 3.4 | 3.6 | 3.6 | 21.8 |

**References**

1.	Liu R, Moss B. 2016. Opposing Roles of Double-Stranded RNA Effector Pathways and Viral Defense Proteins Revealed with CRISPR-Cas9 Knockout Cell Lines and Vaccinia Virus Mutants. J Virol 90:7864-79.

2.	Liu X, Zhang C, Li H. 2024. Neuroinflammation in the medullary visceral zone exert a powerful impaction on the systemic inflammation in sepsis through cholinergic anti-inflammatory pathway. Sci Rep 14:16921.
